# Supplementary material for: The non-coding RNA SVALKA locus produces a cis-natural antisense transcript that negatively regulates the expression of CBF1 and biomass production at normal temperatures
Source: Plant Commun. 2023 Jan 21;4(4):100551. doi: 10.1016/j.xplc.2023.100551 (PMC10363475; doi:10.1016/j.xplc.2023.100551)
Supplement: Document S1. Supplemental Figures 1–4 and Supplemental Tables 1 and 2 [file mmc1.pdf]

Plant Communications, Volume *4*

Supplemental information

The non-coding RNA *SVALKA* locus produces a *cis*-natural antisense transcript that negatively regulates the expression of *CBF1* and bio-mass production at normal temperatures

Vasiliki Zacharaki, Shiv Kumar Meena, and Peter Kindgren

Supplementary Information

Zacharaki et al.

Supplementary Figure 1-4

Supplementary Table 1: AGO1 small RNAs

Supplementary Table 2: Oligos used in this study

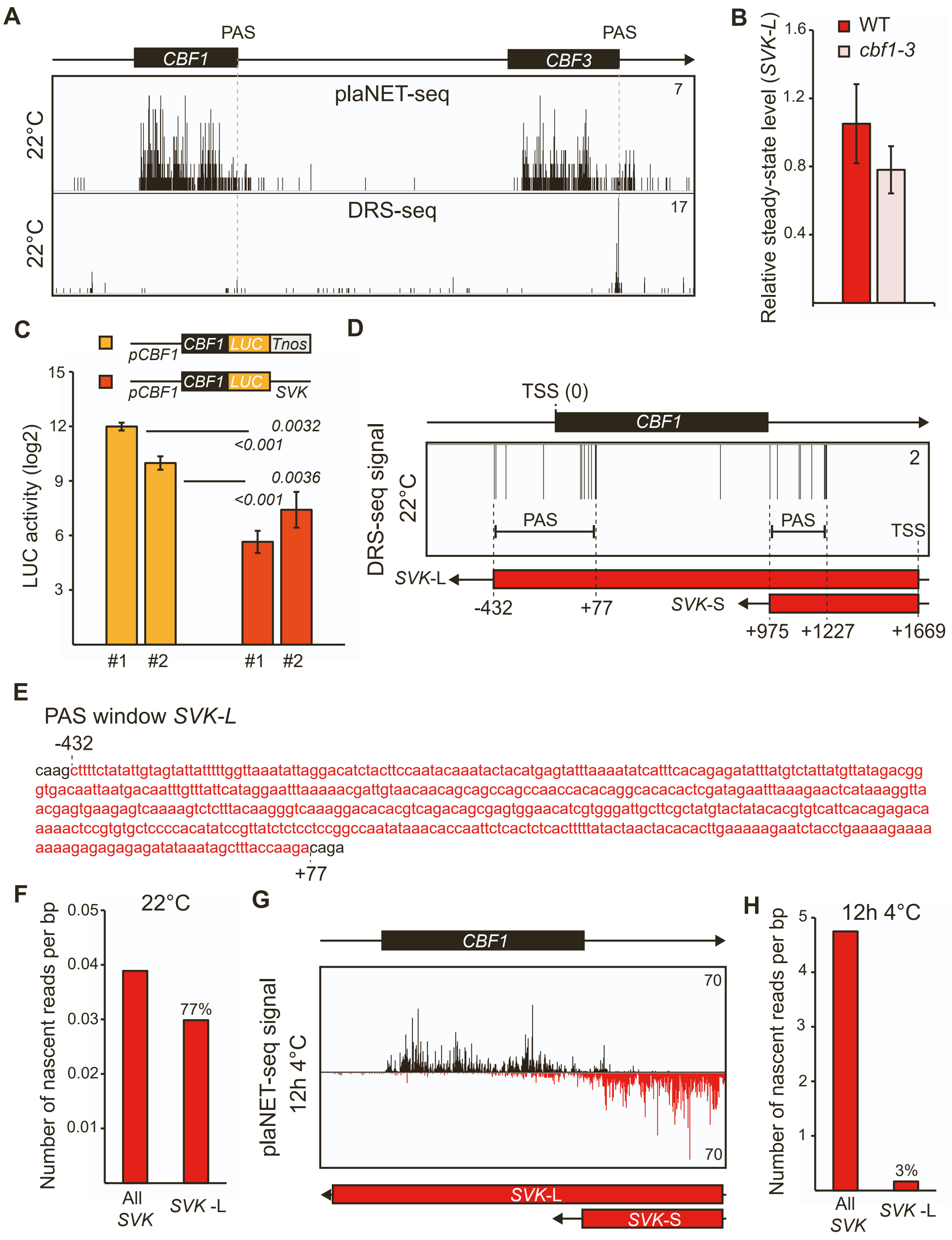
A
PAS
PAS
CBF1
CBF3
22°C
plaNET-seq
7
22°C
DRS-seq
17
B
Relative steady-state level (SVK-L)
1.6
1.2
0.8
0.4
WT
cbf1-3
C
pCBF1 CBF1 LUC Tnos
pCBF1 CBF1 LUC SVK
LUC activity (log2)
15
12
9
6
3
0.0032
<0.001
0.0036
<0.001
#1
#2
#1
#2
D
TSS (0)
CBF1
DRS-seq signal
22°C
2
PAS
PAS
TSS
SVK-L
-432
+77
SVK-S
+975
+1227
+1669
E
PAS window SVK-L
-432
caagcttttctatattgtagtattatttttggttaaatattaggacatctacttccaatacaaatactacatgagtatttaaaatatcatttcacagagatatttatgtctattatgttatagacgg
gtgacaattaatgacaatttgtttattcataggaatttaaaaacgattgtaacaacagcagccagccaaccacacaggcacacactcgatagaatttaaagaactcataaaggtta
acgagtgaagagtcaaaagtctctttacaagggtcaaaggacacacgtcagacagcgagtggaacatcgtgggattgcttcgctatgtactatacacgtgtcattcacagagaca
aaaactccgtgtgctccccacatatccgttatctctcctccggccaatataaacaccaattctcactctcactttttatactaactacacacttgaaaaagaatctacctgaaaagaaaa
aaaagagagagagatataaatagctttaccaagacaga
+77
F
22°C
Number of nascent reads per bp
0.05
0.04
0.03
0.02
0.01
77%
All SVK
SVK -L
G
CBF1
plaNET-seq signal
12h 4°C
70
70
SVK-L
SVK-S
H
12h 4°C
Number of nascent reads per bp
5
4
3
2
1
3%
All SVK
SVK -L

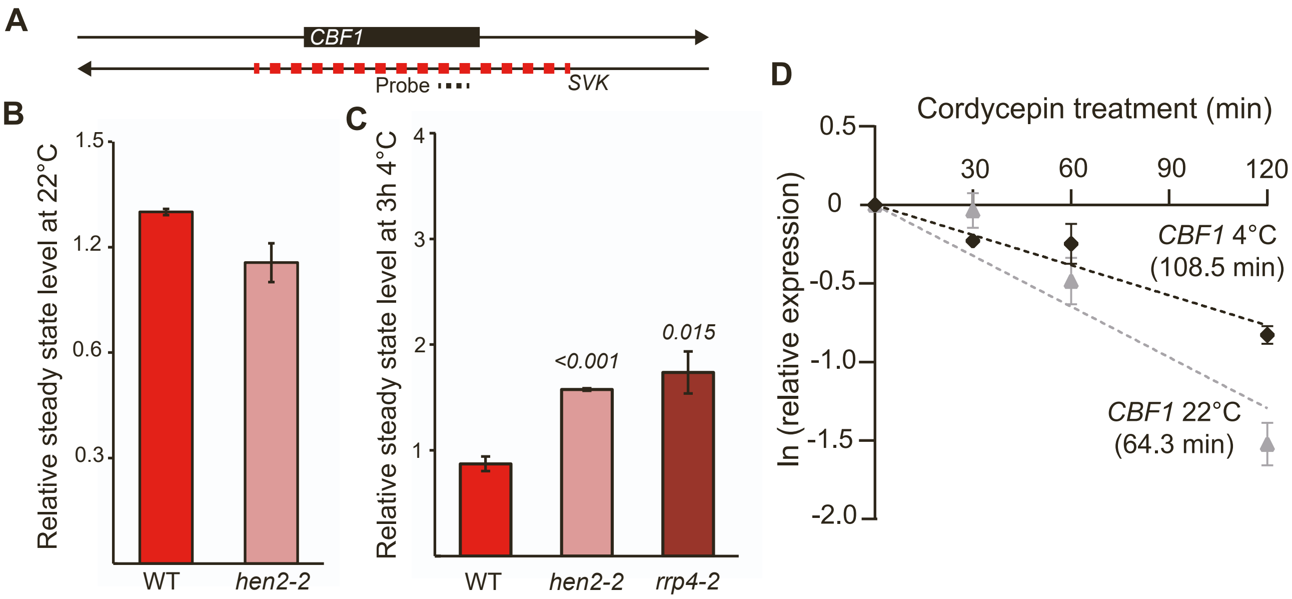
A
CBF1
Probe
SVK
B
Relative steady state level at 22°C
1.5
1.2
0.6
0.3
WT
hen2-2
C
Relative steady state level at 3h 4°C
4
3
2
1
<0.001
0.015
WT
hen2-2
rrp4-2
D
Cordycepin treatment (min)
0.5
0
-0.5
-1.0
-1.5
-2.0
30
60
90
120
ln (relative expression)
CBF1 4°C
(108.5 min)
CBF1 22°C
(64.3 min)

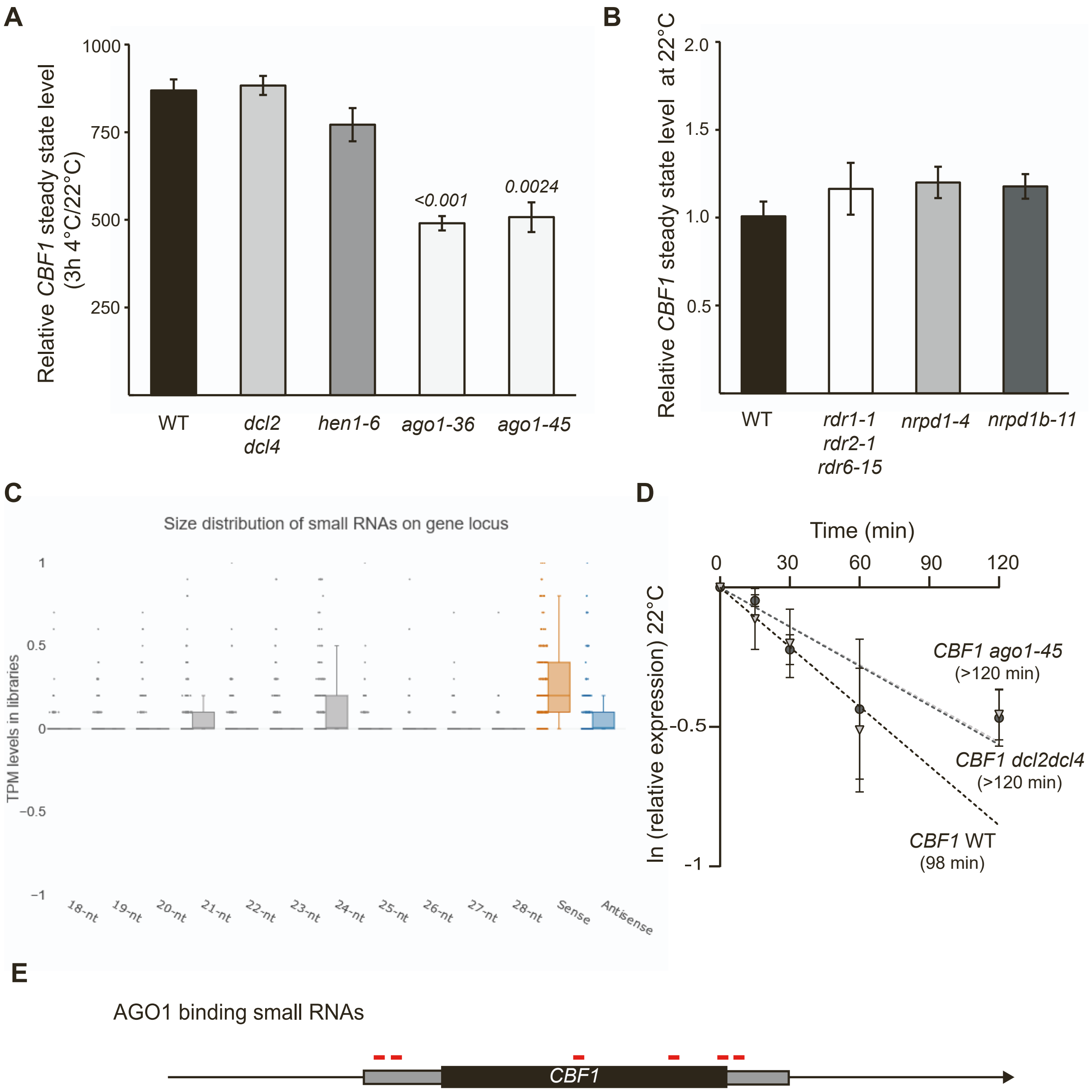

A
Relative CBF1 steady state level (3h 4°C/22°C)
1000
750
500
250
<0.001
0.0024
WT
dcl2 dcl4
hen1-6
ago1-36
ago1-45
B
Relative CBF1 steady state level at 22°C
2.0
1.5
1.0
0.5
WT
rdr1-1 rdr2-1 rdr6-15
nrpd1-4
nrpd1b-11
C
Size distribution of small RNAs on gene locus
TPM levels in libraries
1
0.5
0
−0.5
−1
18-nt
19-nt
20-nt
21-nt
22-nt
23-nt
24-nt
25-nt
26-nt
27-nt
28-nt
Sense
Antisense
D
Time (min)
0
30
60
90
120
ln (relative expression) 22°C
-0.5
-1
CBF1 ago1-45 (>120 min)
CBF1 dcl2dcl4 (>120 min)
CBF1 WT (98 min)
E
AGO1 binding small RNAs
CBF1

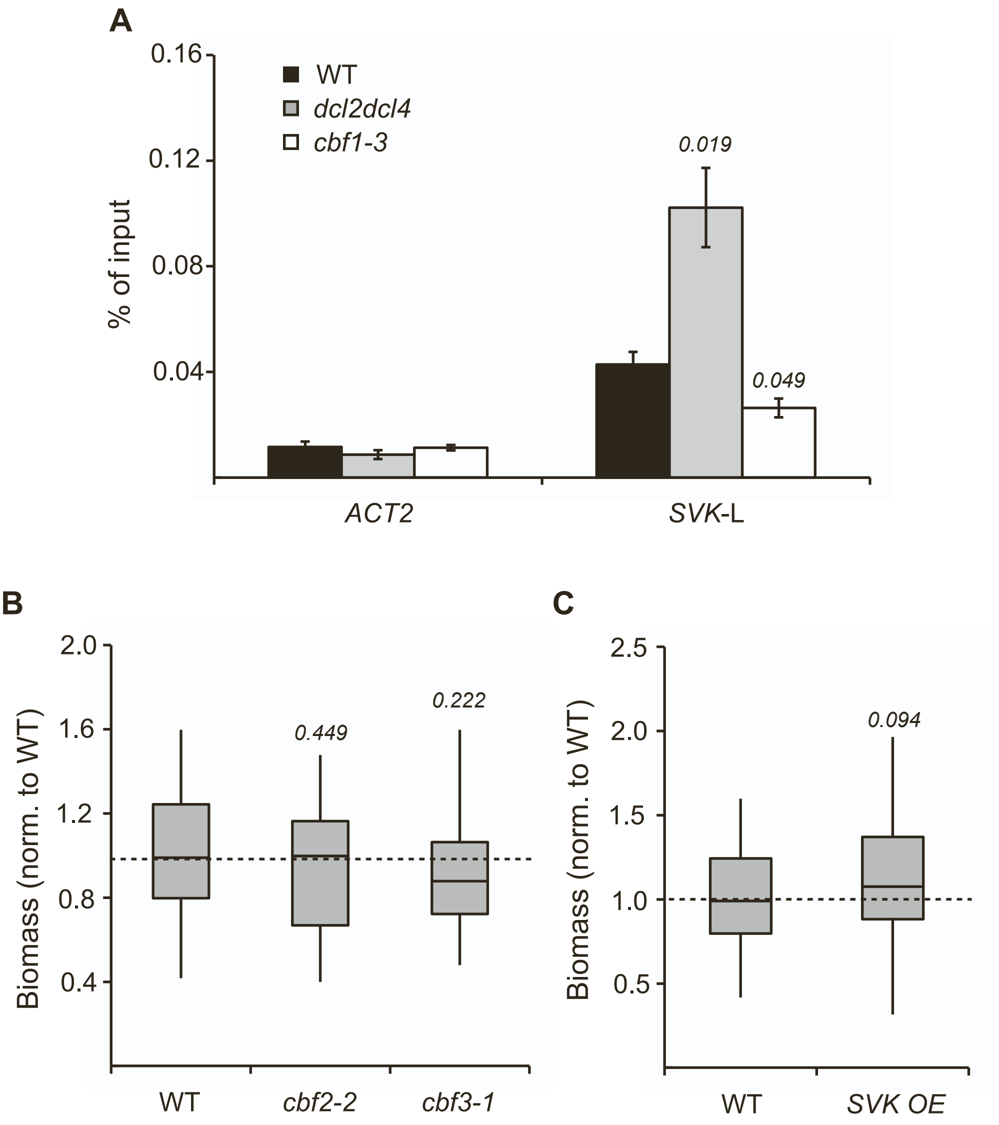

A
0.16
0.12
0.08
0.04
% of input
WT
dcl2dcl4
cbf1-3
0.019
0.049
ACT2
SVK-L
B
2.0
1.6
1.2
0.8
0.4
Biomass (norm. to WT)
0.449
0.222
WT
cbf2-2
cbf3-1
C
2.5
2.0
1.5
1.0
0.5
Biomass (norm. to WT)
0.094
WT
SVK OE

Supplementary Table 1

small RNAs binding to AGO1

**Sequence**
tgaaaaagaatctacc
aaaaaaagagagagagatataaa
ACTTCGCTGACTCGGCTTGGCGGCTACGAATCCCGG
TATATGGATGAGGAGACAATG
GAGTTACTAAtatt
tttccatttttgtactatagt

Supplementary Table 2

Oligos used in this study

For RT-qPCR

Locus	Name	Sequence (5´-3´)	Comment
-	rtTAG	GACTGGAGCACGAGGACACT	Oligo used in qPCR to amplify sequence from linker on RT primer
At4g25490	*CBF1*	GACTGGAGCACGAGGACACggtaaagctatttatatctctctctc	RT primer
		cacacttgaaaaagaatctacctg	Forward primer in qPCR
At4g07395	*SVK (probe c)*	GACTGGAGCACGAGGACACTacccgaaataaacaaatccgta	RT primer
		ggagaagcaagacgacaacg	Forward primer in qPCR
	SVK (probe b)	GACTGGAGCACGAGGACACTtcgatagtcgtttccatttttgt	RT primer
		aaaatgaaggaaccattctaaaaa	Forward primer in qPCR
	SVK (probe a)	GACTGGAGCACGAGGACACTcacacttgaaaaagaatctacctg	Reverse primer in qPCR, RT primer
		ggtaaagctatttatatctctctctc	Forward primer in qPCR
At3g18780	*ACT2*	CTTGCACCAAGCAGCATGAA	Reverse primer in qPCR, RT primer
		CCGATCCAGACACTGTACTTCCTT	Forward primer in qPCR
At4g05320	*UBQ10*	GGCCTTGTATAATCCCTGATGAATAAG	Reverse primer in qPCR, RT primer
		AAAGAGATAACAGGAACGGAAACATAGT	Forward primer in qPCR
At3g45970	*EXP*	CAAGTCGGTTCATCGCCAAATTGGG	Reverse primer in qPCR, RT primer
		GTATCCACCGGTTACTACGAACCTG	Forward primer in qPCR
At3g13920	*EIF4A*	GGCGTAAGGTTGATTGGCTCAC	Reverse primer in qPCR, RT primer
		GATGAGAACACGGGAGGAACCAG	Forward primer in qPCR
