# Supplementary material for: The non-coding RNA SVALKA locus produces a cis-natural antisense transcript that negatively regulates the expression of CBF1 and biomass production at normal temperatures
Source: Plant Commun. 2023 Jan 21;4(4):100551. doi: 10.1016/j.xplc.2023.100551 (PMC10363475; doi:10.1016/j.xplc.2023.100551)
Supplement: Document S2. Article plus supplemental information [file mmc2.pdf]

Plant Communications
Research article

CellPress Partner Journal

The non-coding RNA *SVALKA* locus produces a *cis*-natural antisense transcript that negatively regulates the expression of *CBF1* and biomass production at normal temperatures

Vasiliki Zacharaki, Shiv Kumar Meena and Peter Kindgren*

Umeå Plant Science Centre, Department of Forest Genetics and Plant Physiology, Swedish University of Agricultural Sciences, 90187 Umeå, Sweden

***Correspondence: Peter Kindgren (peter.kindgren@slu.se)**

https://doi.org/10.1016/j.xplc.2023.100551

ABSTRACT

Non-coding transcription is present in all eukaryotic genomes, but we lack fundamental knowledge about its importance for an organism's ability to develop properly. In plants, emerging evidence highlights the essential biological role of non-coding transcription in the regulation of coding transcription. However, we have few molecular insights into this regulation. Here, we show that a long isoform of the long non-coding RNA *SVALKA*-L (*SVK*-L) forms a natural antisense transcript to the host gene *CBF1* and negatively regulates *CBF1* mRNA levels at normal temperatures in the model plant *Arabidopsis thaliana*. Furthermore, we show detailed evidence for the specific mode of action of *SVK*-L. This pathway includes the formation of double-stranded RNA that is recognized by the DICER proteins and subsequent downregulation of *CBF1* mRNA levels. Thus, the *CBF1-SVK* regulatory circuit is not only important for its previously known role in cold temperature acclimation but also for biomass production at normal temperatures. Our study characterizes the developmental role of *SVK*-L and offers mechanistic insight into how biologically important overlapping natural antisense transcripts can act on and fine-tune the steady-state levels of their host gene's mRNA.

Key words: *SVALKA*, *cis*-natural antisense transcript, non-coding transcription, *CBF1*, *Arabidopsis*

Zacharaki V., Meena S.K., and Kindgren P. (2023). The non-coding RNA *SVALKA* locus produces a *cis*-natural antisense transcript that negatively regulates the expression of *CBF1* and biomass production at normal temperatures. Plant Comm. **4**, 100551.

INTRODUCTION

Climate change is increasing global temperatures, and future winters are thus predicted to be milder. This change is harmful to plants, as it disrupts their ability to acclimate to cold temperatures (Shepherd, 2016). The key players in the plant cold acclimation process are the C-repeat binding factors (CBFs) (Thomashow, 1999). Expression of the neighboring genes *CBF1–3* is transiently upregulated within minutes of cold exposure, and their products are responsible for activating the early steps of cold acclimation (Medina et al., 1999). *CBF1*, one of the three cold-induced *CBFs*, has been extensively studied for its role in cold acclimation in a wide range of plant species (Jaglo-Ottosen et al., 1998; Benedict et al., 2006; Ito et al., 2006; Badawi et al., 2007; Zhang et al., 2011).

The role of *CBF1* in cold protection is well established. However, part of its regulation at cold temperatures has only recently been described (Kindgren et al., 2018). The long non-coding RNA (lncRNA) *SVALKA* (*SVK*) is transcribed downstream and from the antisense strand of *CBF1*. *SVK* has the potential to be a *cis*-natural antisense transcript (NAT) if transcription proceeds over the *CBF1* gene body. NATs can form double-stranded RNA with the coding mRNA when both RNAs are present in the same cell. This can be recognized by the RNA silencing machinery and processed into small interfering RNAs (siRNAs) (Bologna and Voinnet, 2014). *cis*-NATs are a common feature of *Arabidopsis* genes. A recent study that adapted native elongation transcript sequencing (plaNET-seq) to *Arabidopsis* found that over 30% of all expressed genes have an associated long non-coding *cis*-NAT (Kindgren et al., 2019). Remarkably, considering that *cis*-NATs have been known for a long time, we know little about their general function (Bologna and Voinnet, 2014). Studied examples of *cis*-NATs have often found idiosyncratic mechanisms with few overlapping components

Published by the Plant Communications Shanghai Editorial Office in association with Cell Press, an imprint of Elsevier Inc., on behalf of CSPB and CEMPS, CAS.

This is an open access article under the CC BY license (http://creativecommons.org/licenses/by/4.0/).

(Zhang et al., 2012, 2013; Yuan et al., 2015; Reis and Poirier, 2021). Still, a common working hypothesis is that *cis*-NATs are especially important in regulating developmental and environmental decisions by the plant (Zhang et al., 2013).

Indeed, at cold temperatures, *SVK* negatively fine-tunes the transcriptional output of *CBF1* via an RNA Polymerase II (RNAPII) collision mechanism due to extreme cold-induced transcriptional activity over the *CBF1* gene body (Kindgren et al., 2018). Collisions occur when *CBF1* expression has reached its peak after 2 to 4 h of cold exposure, resulting in premature termination and rapid degradation of *CBF1* transcripts (Kindgren et al., 2018). This intricate lncRNA-mediated regulation highlights the importance of maintaining output from the *CBF1* gene at a controlled level throughout the cold response.

Interestingly, *CBF1* regulation is not restricted to cold acclimation. *CBF1* is regulated under normal growth conditions by other environmental stimuli, including light, plastid, and circadian signals (Zarka et al., 2003; Fowler et al., 2005; Norén et al., 2016; Jiang et al., 2020). Moreover, the *CBF1* mRNA was shown to be post-transcriptionally regulated and to have the highest turnover of any mRNA in *Arabidopsis* at 22°C (Sorenson et al., 2018). A possible hint about the importance of maintaining finely tuned *CBF1* expression levels comes from the fact that both overexpression and knockdown of *CBF1* result in the same stunted growth phenotype (Jaglo-Ottosen et al., 1998; Dong et al., 2020). Thus, earlier findings pinpoint *CBF1* as a key player under both normal and cold temperatures, although why plants invest considerable amounts of energy maintaining *CBF1* mRNA levels under such tight surveillance is an outstanding question.

In this study, we expand our knowledge of *CBF1* regulation mediated by non-coding *SVK* transcription and its role in plant development and growth. We show that the regulatory cascade of *SVK* antisense transcription on *CBF1* sense transcription is a dynamic process, active at normal temperatures, with a mechanism distinct from that at cold temperatures. At 22°C, *SVK* transcription navigates the antisense strand of *CBF1* to form double-stranded RNA (dsRNA) with the sense mRNA, and this decreases the half-life of the *CBF1* mRNA. Impaired *SVK* expression has a biological role and leads to increased biomass production at 22°C due to mis-regulation of *CBF1*. Our study identifies important players in the *cis*-NAT–host gene regulatory pathway and demonstrates that *CBF1* is a major plant growth regulator.

RESULTS

SVK transcription traverses the *CBF1* gene body at normal temperatures

To understand the mechanisms behind the rapid turnover of *CBF1* at 22°C, we first corroborated the degradation kinetics of the *CBF1* mRNA with an orthogonal technique. We used plant Native Elongation Transcript Sequencing (plaNET-seq) data (Kindgren et al., 2019) over *CBF1* and its neighboring gene *CBF3* coupled with direct RNA-sequencing data (DRS). DRS detects stable polyadenylated RNAs (Supplemental Figure 1A), whereas plaNET-seq detects nascent actively transcribed RNAs. plaNET-seq showed that *CBF1* and *CBF3* were actively transcribed at similar levels. However, DRS-seq showed that *CBF3* had a prominent peak at the predicted poly(A) (polyadenylation) site, whereas *CBF1* barely had any detectable peak. This suggests that *CBF1* is highly transcribed, but its mRNA is rapidly degraded at 22°C compared with the *CBF3* mRNA (Supplemental Figure 1A). A possible explanation is the presence of an antisense transcript derived from transcription of the lncRNA *SVALKA (SVK)*. Indeed, we observed extensive *SVK* transcription throughout the *CBF1* gene body at 22°C using the plaNET-seq data (Figure 1A). Low transcriptional activity in the region would decrease the probability of RNAPII collisions at 22°C compared with 4°C (Kindgren et al., 2018). Thus, if *SVK* has any regulatory potential to fine tune the expression levels of *CBF1* at 22°C, it is likely to involve a mechanism distinct from that of *CBF1-SVK* regulation at 4°C.

To investigate the role of *SVK* at 22°C, we isolated a line that harbors a transfer DNA (T-DNA) insertion in the *CBF1* promoter, which limits *CBF1* expression without affecting steady-state levels of *SVK* (*cbf1-3*, Figure 1B–1D, Supplemental Figure 1B). In T-DNA lines, large inserts of DNA disrupt the genomic context of the insertion site (Alonso et al., 2003). We also included the *uns-1* T-DNA mutant that uncouples the expression of *CBF1* and *SVK*, and the *svk-1* T-DNA mutant that knocks out *SVK* (Figure 1B–1D) (Kindgren et al., 2018). Interestingly, *CBF1* steady-state levels increased in the *uns-1* and *svk-1* mutants (Figure 1C), indicating that *SVK* may indeed have a regulatory role toward *CBF1* at 22°C. *SVK* steady-state levels were unaffected in *cbf1-3* and increased in *uns-1* (Figure 1D). The common feature of *svk-1* and *uns-1* is that *SVK* transcription cannot proceed into the *CBF1* gene body in both mutants (Kindgren et al., 2018). To further confirm a *cis* role for *SVK* at 22°C, we used firefly *LUCIFERASE* (*LUC*) reporter constructs. In one construct ($_{p}$*CBF1::CBF1-LUC-T*$_{NOS}$), *LUC* was fused between the coding sequence of *CBF1* and the T_{NOS} terminator. The T_{NOS} terminator was used to abolish any antisense transcription (Kindgren et al., 2018). In the other construct ($_{p}$*CBF1::CBF1-LUC-SVK*), *LUC* was fused between *CBF1* and the endogenous *SVK* sequence, enabling *SVK* transcription along the *LUC* gene body (Kindgren et al., 2018). We used homozygous stable transgenic lines from two independent transformation events for each construct. LUC activity was lower in lines that included *SVK* than in those with the T_{NOS} terminator (Supplemental Figure 1C). Thus, these results strongly suggest that *SVK* transcription along the *CBF1* gene body negatively regulates the *CBF1* gene output at 22°C.

The main 5′ end of *SVK* at 22°C is situated 1669 base pairs (bp) downstream of the transcription start site of *CBF1* (Kindgren et al., 2018). To further characterize the long isoform of *SVK* (*SVK*-L) at 22°C, we used DRS-seq to detect the 3′ end of the transcript (Supplemental Figure 1D). We found two poly(A)-signal intervals, one proximal for the short *SVK* isoform (*SVK*-S) that is dominant at 4°C (Kindgren et al., 2018) and a longer distal poly(A) interval that ends close to the 5′ end of *CBF1* (−432 to +77 with respect to the TSS of *CBF1*, Supplemental Figure 1D and 1E). Based on nascent transcription data, we estimated that about 75% of *SVK* transcription extends to the distal poly(A) signal at 22°C (Supplemental Figure 1F), a result that is consistent with the level of poly(A) transcripts detected by DRS-seq. By contrast, after 12 h at 4°C, nascent transcription levels indicated that only about 3% of *SVK* transcription extends

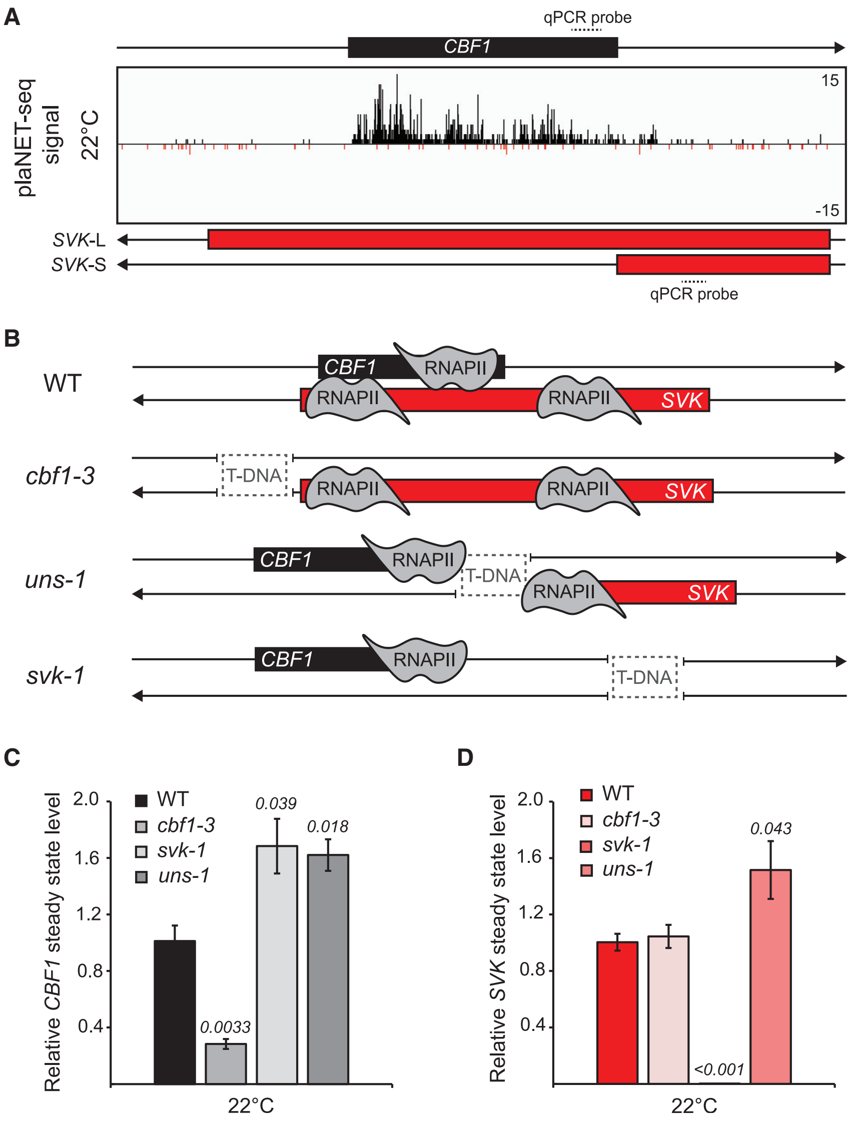

Figure 1. A long isoform of *SVK* is present at 22°C.

(A) plaNET-seq screenshot of the *CBF1-SVK* genomic region at 22°C. Nascent RNAPII sense transcription is shown in black and antisense transcription in red.

(B) Graphical illustrations of the positions of T-DNA insertions in the investigated genotypes and their effects on *CBF1* and *SVK* transcription.

(C and D) The relative steady-state level of **(C)** *CBF1* and **(D)** *SVK* measured with RT-qPCR at 22°C. Steady-state levels were normalized to the levels observed in the wild type. The mean values were derived from three biological replicates. Error bars represent ±SEM. Statistical significance was calculated with Student's t test. p values are shown in the figure.

to the distal poly(A) signal (Supplemental Figure 1G and 1H). This corroborates earlier results from 4°C (Kindgren et al., 2018) and indicates that *SVK*-L is the dominant isoform of *SVK* at 22°C.

Our results demonstrate that *SVK* transcription negatively regulates *CBF1* mRNA levels and that *SVK*-L is predominant at 22°C and may be involved in *CBF1* regulation.

SVK-L modulates *CBF1* mRNA stability

At 4°C, readthrough transcription from the dominant proximal poly(A) site of *SVK* is quickly degraded by the nuclear exosome (Kindgren et al., 2018). To investigate whether *SVK*-L is under nuclear exosome surveillance at 22°C, we measured the steady-state level of *SVK* with two qPCR probes in the wild type and the *rrp4-2* mutant. (Figure 2A). RRP4 is an exosome core subunit and is present in both nuclear and cytosolic exosome complexes (Lange et al., 2014). Both probes showed an increased *SVK* level in *rrp4-2*, indicating that it is an exosome target at 22°C (Figure 2B and 2C). Interestingly, and in contrast to 4°C, *SVK* is not a HEN2 target at 22°C (Supplemental Figure 2A–2C). HEN2 is an exosome subunit that associates only with the complex in the nucleoplasm (Lange et al., 2014). This suggests that RNA products of *SVK* transcription along the *CBF1* gene body are degraded by exosome complexes with distinct compositions in a temperature-dependent manner. The intriguing degradation kinetics of *CBF1* and *SVK* led us to investigate their half-lives with a cordycepin assay (Fedak et al., 2016). Cordycepin inhibits transcription, and after its incorporation during RNA synthesis, it is possible to follow the degradation of specific transcripts. In wild-type seedlings, *CBF1* displayed a half-life of 97.5 ± 2.5 min and *SVK* of 66.7 ± 3.8 min (Figure 2D). These values fall between the half-life of the stable mRNA of *EUKARYOTIC INITIATION FACTOR 4A-1* (*EIF4A1*) (>120 min) and the highly unstable transcript of *EXPANSIN L1* (*EXPL1*, 51 ± 9.6 min), which were used as assay controls. Our *cbf1-3* and *svk-1* mutants allowed us to further test our hypothesis that *CBF1* degradation depends on the presence of *SVK* and vice versa. Indeed, blocking *SVK* transcription (*svk-1*) resulted in higher stability of *CBF1* (Figure 2E). Conversely, downregulation of *CBF1* (*cbf1-3*) resulted in a more stable *SVK* (Figure 2F). We could also confirm that *SVK*-L is an RRP4 target at 22°C (Figure 2F). Interestingly, *CBF1* stability is increased at 4°C, suggesting that *SVK* is not responsible for degradation of full-length *CBF1* mRNA at cold temperatures (Supplemental Figure 2D). These results are compelling evidence that the presence of *SVK*-L *cis*-NAT is responsible for degrading *CBF1* mRNA at 22°C but not at 4°C. Furthermore, they show that the abundances of both transcripts depend on each other's presence and absence, supporting the idea that *SVK*-L and *CBF1* transcripts may undergo hybridization *in planta*, leading to the formation of double-stranded RNA (dsRNA) at 22°C.

CBF1-SVK forms dsRNA at 22°C

According to a consensus model, long dsRNA templates formed by a sense mRNA-*cis*-NAT are recognized as substrates by the

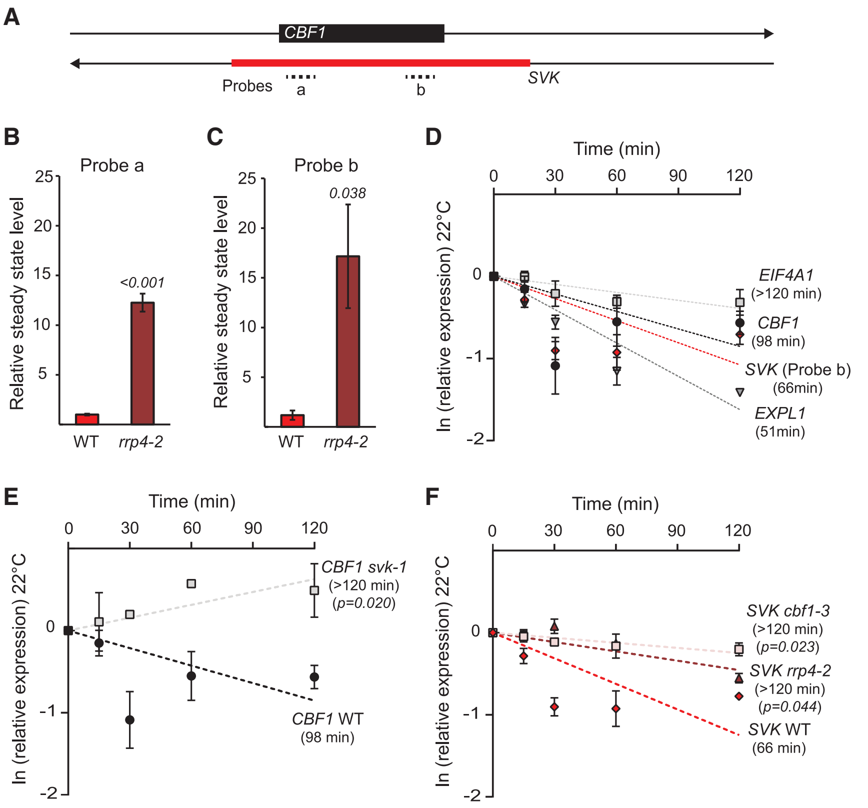

Figure 2. The transcript stabilities of *SVK* and *CBF1* depend on each other's presence.

(A) Graphical illustration of probes on the *SVK* transcription unit.

(B and C) The relative steady-state level of **(B)** probe a and **(C)** probe b measured with RT-qPCR in the wild type (WT) and *rrp4-2* at 22°C. Steady-state levels were normalized to WT levels. The mean values are from three biological replicates. Error bars represent ±SEM. Statistical significance was calculated with Student's t test. p values are shown in the figures.

(D–F) Transcript stability assays for *SVK* and *CBF1* in **(D)** Col-0 (WT), **(E)** *svk-1*, and **(F)** *cbf1-3* and *rrp4-2* seedlings after transcriptional inhibition with cordycepin at 22°C. Half-life ($t_{1/2}$) was determined from the slope of degradation curves that were obtained after RT-qPCR analysis of cordycepin-treated seedlings at the indicated time points. Stable *EUKARYOTIC TRANSLATION INITIATION FACTOR 4A1* (*EIF4A1*) and *EXPANSIN L1 (EXPL1)* are shown as controls in **(D)**. Each data point is the mean of three biological replicates. Error bars represent ±SD. p values for statistical tests of differences in slope are shown in the figures.

endonuclease DICER-LIKE (DCL) proteins, leading to generation of short dsRNA fragments as a result of cleavage activity. The cleavage product(s) are stabilized by HUA ENHANCER 1 (HEN1)-mediated methylation (Figure 3A) (Bologna and Voinnet, 2014), and one of the guide RNA strands from the short dsRNA can be loaded onto ARGONAUTE1 (AGO1) and/or AGO4 to form RNA-induced silencing complex (RISC). RISC may eventually establish a self-perpetuating silencing loop that can potentially amplify the extent of downregulation of the target transcript (Bologna and Voinnet, 2014). Among mutants of the four DCL proteins present in *Arabidopsis*, only *dcl2dcl4* displayed higher *CBF1* expression at 22°C (Figure 3B). Similarly, *hen1* showed increased *CBF1* mRNA levels, implicating several components in the regulation of *CBF1* at 22°C (Figure 3C). We also observed increased *CBF1* mRNA levels in two separate *ago1* mutants but not in an *ago4* mutant (Figure 3D), suggesting specific involvement of AGO1 in the regulation of *CBF1* expression at 22°C. In line with our previous results, none of the mutants that had increased *CBF1* expression levels at 22°C showed any mis-regulation of *CBF1* after cold induction, suggesting that the DCL pathway is not active at 4°C when *CBF1* needs to be rapidly induced (Supplemental Figure 3A). To further investigate whether the RNA-directed DNA methylation (RdDM) pathway is active in *CBF1* transcriptional gene silencing via small RNAs (sRNAs), we measured *CBF1* mRNA levels in mutants of the plant-specific RNA polymerases Pol IV and Pol V. However, *CBF1* expression in both *pol IV* and *pol V* mutants was similar to wild-type levels at 22°C (Supplemental Figure 3B), reinforcing the plaNET-seq data and pointing to the conclusion that *CBF1* downregulation is most likely exclusively dependent on RNAPII transcription. To determine whether cleavage products generated after DICER activity from the *CBF1*:*SVK*-L hybrid are subject to sequential amplification by *RNA DEPENDENT RNA POLYMERASE* (*RDR*) *1*, *2*, or *6* at 22°C, we quantified transcript abundance of *CBF1* in the *rdr 1*, *2*, *6* triple mutant (*rdr1-1*, *rdr2-1*, *rdr6-15*). Our results suggested that RDRs are not part of this regulation, based on wild-type levels of *CBF1* in the mutants (Supplemental Figure 3B). In addition, we mined the *Arabidopsis* Small RNA Database (Feng et al., 2019) to see whether there was an accumulation of sRNAs in the *CBF1* region. There was a small accumulation of sRNAs 21 nucleotides (nt) and 24 nt in length, indicating that sRNAs are present on the *CBF1* locus but at a low level (Supplemental Figure 2C). Together, these results suggest that the *CBF1::SVK*-L dsRNA does not lead to anticipated gene silencing amplification from other RNA polymerases, supporting the presence of a stringent regulatory landscape for *CBF1* expression that allows only its fine-tuning rather than complete silencing, making the cleavage products inherently challenging to detect with currently available methods.

Nevertheless, to further corroborate our hypothesis, we performed RNase protection assays that detect local dsRNA (Okano et al., 2014). We used three probes on the *SVK*-L transcription unit (Figure 4A), and we could confirm an increased *CBF1* level in *dcl2dcl4* without RNase treatment (Figure 4B). After RNase treatment, we could detect a signal for dsRNA only at probe b in the 3′ end of *CBF1* in the wild type (Figure 4C and 4D). The signal was significantly higher in the *dcl2dcl4* mutant, indicating that dsRNAs were formed in the region and recognized as substrates for DCL2 and/or DCL4, leading to their degradation. Supporting these results, we also detected a longer half-life for the *CBF1* mRNA in both *dcl2dcl4* and *ago1-45* mutants, further suggesting their direct involvement in *CBF1* degradation (Supplemental Figure 3D). To confirm the

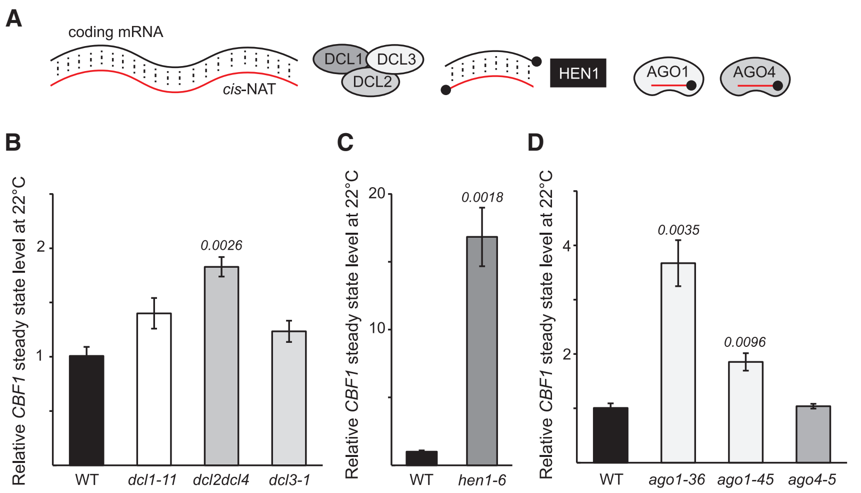

Figure 3. The RNA silencing pathway degrades *CBF1* mRNA.

(A) Graphical illustration of the proposed players in the *cis*-NAT pathway.

(B–D) The relative steady-state level of *CBF1* measured by RT-qPCR in different mutants involved in RNA silencing at 22°C. Steady-state levels were normalized to wild-type (WT) levels. The mean values are from three biological replicates. Error bars represent ±SEM. Statistical significance was calculated with Student's t test. p values are shown in the figures.

direct involvement of AGO1, we used AGO1-IP RNA data to identify potential AGO1-bound RNAs. Several RNAs were found in the *CBF1* region (Supplemental Figure 3E), Supplemental Table 1). Intriguingly, we could detect three 21-nt AGO1-bound RNAs in the 3′ half of *CBF1*, corresponding to the region where we detected the dsRNAs. All in all, these results demonstrate that several components are directly involved in degradation of *CBF1::SVK*-L dsRNA hybrids at 22°C, confirming the regulation of *CBF1* by *SVK* during normal development. The dsRNA formation also strongly suggests that *SVK*-L achieves this downregulation in a *cis* manner.

SVK-CBF1 regulation governs biomass production at 22°C

To better understand the regulation of *CBF1 mRNA by SVK*-L and its biological role, we monitored *CBF1* steady-state levels during the light period in our *cbf1* and *svk* mutants under standard short-day conditions (Figure 5A). In line with earlier reports, *CBF1* levels peaked shortly after the middle of the day (Norén et al., 2016). Steady-state *CBF1* levels in *svk-1* and *uns-1* plants increased at time points close to the end of the day (Figure 5A). As expected, steady-state levels of *CBF1* were lower in the *cbf1-3* mutant throughout the day (Figure 5A). Intriguingly, the *SVK* steady-state level peaked at the end of the light period, showing a similar anti-correlation to *CBF1* expression during cold exposure (Figure 5B) (Kindgren et al., 2018). Both *cbf1-3* and *uns-1* displayed wild-type levels of *SVK*, whereas *svk-1* had low levels of *SVK* throughout the day (Figure 5B). To confirm that we could also detect dsRNA under these conditions, we performed dsRNA-IP-qPCR using an antibody specific to dsRNA (Supplemental Figure 4A). Here, *dcl2dcl4* again showed a higher level of dsRNA accumulation compared with the wild type. The *cbf1-3* mutant showed lower levels of dsRNA, providing further evidence that dsRNA was formed by *CBF1* and *SVK*-L transcripts and that DCL2 and/or DCL4 are involved in its cleavage. Importantly, we observed a notable difference in biomass production of the mutants, indicating a physiological role for *CBF1-SVK*-L regulation at 22°C (Figure 5C and 5D). In particular, *cbf1-3* showed decreased biomass compared with wild-type plants, whereas *svk-1* and *uns-1* had increased biomass. By contrast, despite their high sequence similarity, we did not observe a similar phenotype for mutants of *CBF1* neighboring genes *CBF3* and *CBF2,* highlighting the absence of full redundancy between the *CBFs* and suggesting a potential *CBF1*-specific role in biomass control (Supplemental Figure 4B). Furthermore, overexpression of *SVK* in *cis* (Kindgren et al., 2018) did not alter biomass production (Supplemental Figure 4C), indicating that *SVK* transcription in wild-type plants is not the limiting factor in this regulation.

In conclusion, our data indicate that fine-tuning the steady-state levels of *CBF1* mRNA is important for regulating biomass production and, in this regulation, the lncRNA *SVK*-L has an intricate *cis*-NAT role in maintaining the proper *CBF1* mRNA level.

DISCUSSION

Our findings from this study can be summarized in a working model of *CBF1* regulation by *SVK* (Figure 6). At normal growing temperatures, *SVK*-L is the dominant isoform of *SVK,* and it functions as a *cis*-NAT that interacts with the host gene's mRNA to form dsRNA. *CBF1-SVK*-L dsRNA is recognized by DCL2 and/or DCL4 and cleaved. The cleaved product is loaded onto AGO1 for further cleavage of *CBF1* mRNA. By contrast, at 4°C, increased transcriptional activity on the *CBF1* gene body limits the presence of *SVK*-L. In addition, a switch to a proximal poly(A) site makes *SVK*-S the major isoform at 4°C. *SVK* transcription is also induced a few hours after *CBF1* during cold exposure, which results in RNAPII collisions and prematurely terminated *CBF1* transcription. Thus, the lncRNA *SVK* negatively downregulates *CBF1* with two distinct and temperature-specific mechanisms.

The transcription factor CBF1 has been extensively studied in plant tolerance to cold and freezing temperatures (Jaglo-Ottosen et al., 1998; Benedict et al., 2006; Ito et al., 2006; Badawi et al., 2007; Zhang et al., 2011). Here, we expand the functional repertoire of CBF1 by demonstrating its significant role in controlling biomass production in a standard growing regimen. A modest increase in *CBF1* mRNA levels results in larger plants (Figure 5), whereas highly increased (Gilmour et al., 2004) or strongly decreased *CBF1* expression leads to lower biomass (Figure 5). Thus, a slightly elevated level of *CBF1* mRNA is beneficial for the plant in a controlled environment, both in terms of biomass production (Figure 5) and cold acclimation (Kindgren et al., 2018). Our results recognize *CBF1* as a converging component in plant development and stress response. Earlier reports place this phenotype in a different

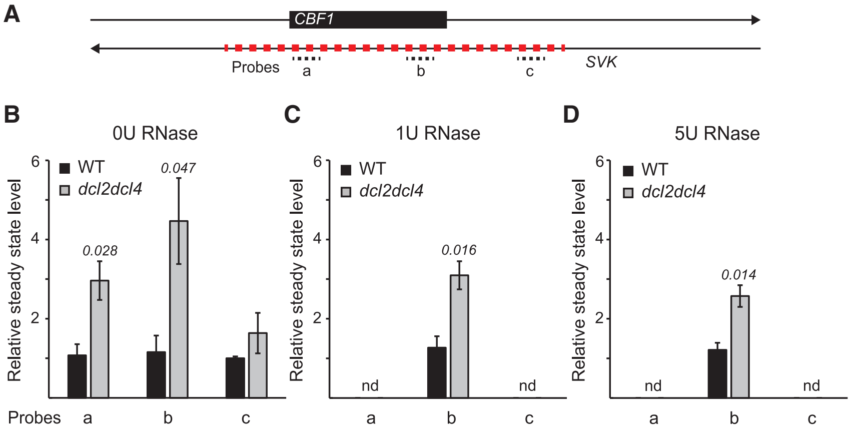

Figure 4. Double-stranded RNA (dsRNA) is formed in the 3′ end of the *CBF1* mRNA.

(A) Graphical illustration of probes on the *SVK* transcription unit.

(B–D) The relative steady-state level of dsRNA in the wild type (WT) and *dcl2dcl4* for **(B)** untreated RNA, **(C)** samples treated with 1U RNase, and **(D)** samples treated with 5U RNase measured with RT-qPCR. Steady-state levels were normalized to the levels observed in untreated samples. The mean values were derived from three biological replicates. Error bars represent ±SEM. Statistical significance was calculated with Student's t test. p values are shown in the figures.

context: light quality regulates *CBF1* and its downstream targets *PHYTOCHROME INTERACTING FACTOR 4* (*PIF4*) and *PIF5,* which form a regulatory hub that controls many processes during plant development and growth (Nieto et al., 2015; Dong et al., 2020). Moreover, plants overexpressing *PIF4* show striking phenotypic similarities to *CBF1*-overexpressing plants (Gangappa et al., 2017). CBF1 is also known to regulate levels of the plant hormone gibberellin. Gibberellin controls plant growth by regulating DELLA proteins (Achard et al., 2008). All these data together highlight the need for tight regulation of *CBF1* mRNA levels in plants to achieve a proper regulatory balance between maximizing biomass production and priming the low-temperature stress responses.

Regulation of *CBF1* involves two distinct temperature-dependent *cis* mechanisms, in which transcription of the lncRNA *SVK* (Kindgren et al., 2018) has a central role. Within minutes of exposure to cold temperatures, *CBF1* expression is quickly induced by transcriptional activators such as ICE1 and CAMTA3 (Zarka et al., 2003; Doherty et al., 2009), which in turn dramatically increases the transcription and steady-state level of *CBF1* to initiate the cold acclimation process (Medina et al., 1999). Massive transcriptional activity on *CBF1* poses a steric hindrance to any RNAPII complex transcribing the antisense strand. However, after 2–3 h of cold treatment, *SVK* transcription is induced, which results in increased accumulation of RNAPII at the 3′ end of *CBF1* (Kindgren et al., 2018). Therefore, the RNAPII collisions that occur during cold exposure require massive transcriptional activity on both strands. By contrast, at normal growing temperatures (22°C), *SVK* transcription does not face the same steric hindrance from sense *CBF1* transcription and gives rise to antisense transcription on the *CBF1* locus (Figure 1). *SVK*-L forms a *cis*-natural antisense transcript (*cis*-NAT) to *CBF1* and triggers the rapid degradation of the *CBF1* mRNA (Figures 2, 3, and 4).

NATs are an important class of lncRNAs with a broad range of molecular functions in eukaryotes (Lapidot and Pilpel, 2006; Britto-Kido et al., 2013). In *Arabidopsis*, the *cis*-NAT *FLORE* is involved in circadian regulation of its host gene, *CYCLING DOF FACTOR 5* (*CDF5*), albeit through an unknown mechanism (Henriques et al., 2017). Another example is the *cis*-NAT$_{PHO1;2}$ in rice, which promotes translation of its host gene, *PHOSPHATE1:2* (Jabnoune et al., 2013). Recent advances suggest that up to 70% of all mRNAs in *Arabidopsis* have an NAT (Wang et al., 2014), and many genes with an NAT are responsive to stress or play developmental roles (Wunderlich et al., 2014; Thieffry et al., 2020; Meena et al., 2021). This suggests an important role for non-coding transcription in plant survival under unfavorable conditions. The sense mRNA and NAT can form dsRNA owing to their sequence complementarity, similar to the first described report in the case of salt stress response in *Arabidopsis* (Borsani et al., 2005). We confirmed that *CBF1* and *SVK*-L also form dsRNA at the 3′ end of *CBF1* (Figure 4, Supplemental Figure 4A). Plants have a highly evolved surveillance system that recognizes dsRNA, which includes four specialized Dicer-like RNase-III endonucleases (DCL1–4) (Bologna and Voinnet, 2014). By contrast, humans (*Homo sapiens*) and worms (*Caenorhabditis elegans*) only have one DCL protein. The *CBF1*-*SVK*-L dsRNA is recognized by DCL2 and/or DCL4 (Figures 3 and 4, Supplemental Figure 4A), two proteins that have been extensively studied during virus infection (Bouché et al., 2006; Deleris et al., 2006). The products of DCL2/4 cleavage are modified (a methyl group is added to the 2′ OH of the RNA) by HEN1 (Figure 3). DCL2/4 and HEN1 are considered to be part of the *cis*-NAT degradation pathway (Bologna and Voinnet, 2014). However, *CBF1*-*SVK*-L dsRNA regulation is not amplified by any RNA DEPENDENT RNA POLYMERASES (RDRs) (Supplemental Figure 3B). One of the RNA strands from the produced short dsRNA is subsequently loaded onto AGO1 for degradation of the *CBF1* mRNA (Figure 3D). Moreover, the *CBF1*-*SVK*-L pathway seems to be specific for AGO1 and does not include AGO4 (Figure 3D). Thus, how specificity is achieved for the DCL and AGO proteins in different *cis*-NAT pathways is an interesting question for future research. The process of *cis*-NAT regulation is complex, and each case often seems idiosyncratic, but our results provide a potential candidate for further studies to unravel this complexity. An important point is that *CBF1*-*SVK*-L regulation does not depend on regulatory enhancement by polymerases other than RNAPII. This may enable careful fine-tuning of *CBF1* in contrast to pathways that include POLIV/POLV and/or RDRs, which would require additional levels of regulation. As a result, DICER generated sRNAs (Supplemental Figure 3C) may be scarce, explaining the finely tuned regulatory window and the difficulty of detecting sRNA products from *cis*-NATs (Wang et al., 2005; Zhang et al., 2012; Li et al., 2013).

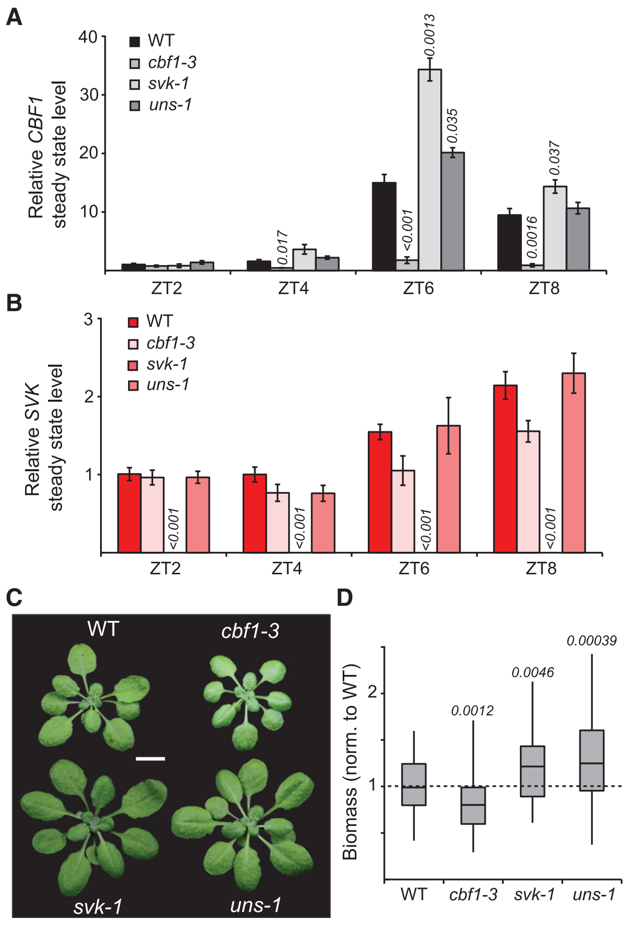

Figure 5. The *CBF1-SVK*-L circuit has a biological role at 22°C.

(A–B) Relative steady-state levels of **(A)** *CBF1* and **(B)** *SVK* were measured through time with RT-qPCR during the light period in the wild type (WT), *cbf1-3*, *svk-1*, and *uns-1*. Steady-state levels were normalized to the levels observed at zeitgeber time 2 (ZT2). The mean values were derived from three biological replicates. Error bars represent ±SEM. Statistical significance was calculated with Student's t test. p values are shown in the figures.

(C) Plants grown under short-day controlled conditions for 4 weeks. Scale bar, 1 cm.

(D) Quantification of biomass of 4-week-old plants grown under short days ($n = 50$). Values have been normalized to the WT biomass. Statistical significance of differences between WT and mutants was calculated with Student's t test, and the p-values are presented in the graph.

Another interesting aspect of this study is the promotion of a dominant poly(A) site for *SVK* approximately 100 bp downstream of the poly(A) site of *CBF1* at 4°C compared with the distal poly(A) site close to the *CBF1* TSS at 22°C (Figure 1) (Kindgren et al., 2018). Alternative polyadenylation (APA) is a common mechanism in eukaryotes for increasing the number of active isoforms derived from one gene (Tian and Manley, 2017). Interestingly, APA also seems to be important for the functional role of lncRNAs. Another temperature switch in poly(A) site preference has been described for the lncRNA *COOLAIR* and its regulation of flowering (Swiezewski et al., 2009). The preferred poly(A) site for *COOLAIR* is controlled by specific RNA 3′-end processing factors (Liu et al., 2010), which could also be the case for *SVK*. However, there is also the possibility that the switch between the main poly(A) site for *SVK* is governed by the increased termination process at the end of the *CBF1* gene body after cold treatment and the fact that *SVK* and *CBF1* are transcribed at the same locus (a requisite for RNAPII collisions), in contrast to *FLC* and *COOLAIR* that are transcribed at mutually exclusive loci (Rosa et al., 2016). Polyadenylation was shown to be promoted in *Arabidopsis* by formation of liquid-liquid phase compartments at the 3′ end of genes (Fang et al., 2019). The distance between the *CBF1* and *SVK*-S poly(A) sites is within the size of these compartments (Fang et al., 2019). Massive transcriptional activity on the *CBF1* gene body early in the cold response would result in the presence of high concentrations of polyadenylation-associated factors in the liquid-liquid phase compartments at the 3′ end of *CBF1*, which would encompass the *SVK*-S poly(A) site. Thus, this presents an elegant way to switch poly(A) preference without the need for additional components. However, future experiments are required to fully understand the poly(A) signal preference we detect for *SVK*.

In this study, we found a connection between 3′–5′ exosome-mediated RNA surveillance and *CBF1-SVK* regulation. The polyadenylated *SVK* transcripts are targeted by the exosome, as seen by a higher transcript level in the *rrp4-2* mutant (Figure 2B and 2C). *SVK* transcripts degraded by the exosome are most likely RNA species that are not part of dsRNA formation or RNA molecules left over after dsRNA cleavage. However, *SVK* transcripts prematurely terminated by RNAPII collisions at 4°C undergo a more rapid degradation (Kindgren et al., 2018). Although all *SVK* transcripts are targeted by the exosome core subunit RRP4, only the prematurely terminated and readthrough transcripts are targeted by exosome complexes that also contain HEN2. In contrast to RRP4, which localizes in both nucleoplasm and cytoplasm, HEN2 is only present in the nucleoplasmic exosome, where active transcription occurs (Lange et al., 2014). This indicates that *SVK* transcripts are degraded by the exosome at spatially separated sites. *Arabidopsis* has two RNA helicases that activate the nuclear exosome, HEN2 and the rRNA processing helicase AtMTR4 (Lange et al., 2014). HEN2 is a plant-specific protein, whereas MTR4 is an essential RNA helicase for all nuclear exosome activities in yeast and humans (Bernstein et al., 2008; Lubas et al., 2011). Our results suggest that prematurely terminated transcripts are degraded in a HEN2-dependent manner close to the active RNA synthesis site compared with transcripts that have reached their poly(A) signal. This is true for both coding and non-coding transcripts. Interestingly, the involvement of HEN2 in the *CBF1-SVK* circuit might suggest that sessile plants have evolved specialized nuclear exosome RNA helicases to properly respond to stress stimuli.

Our study places *CBF1* as a key component in many developmental decisions made by the plant. The extreme regulatory pressure on *CBF1* and its mRNA presented here and in other studies allows for little buffering capacity and demands a high level of fine-tuning. This complex regulation is partially achieved by the lncRNA *SVK* and its different modes of action, providing an impressive rapid and temperature-dependent flexibility for determining the steady-state level of *CBF1* mRNA .

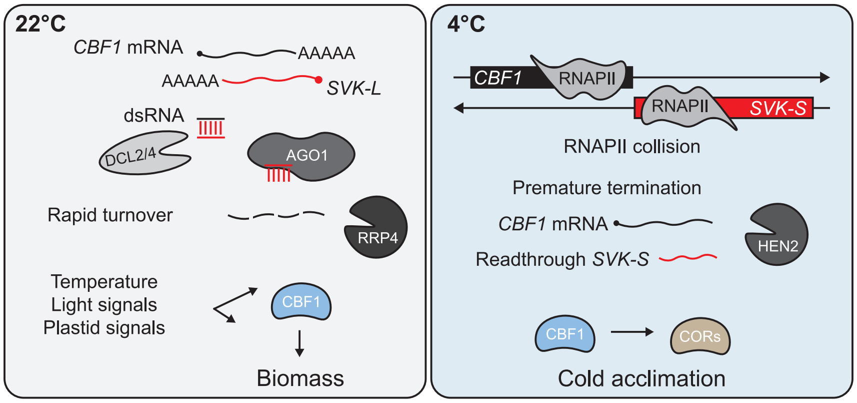

Figure 6. Working model of *CBF1-SVK* regulation.

Model of downregulation of *CBF1* by *SVK* at 22°C and 4°C. *CBF1* and *SVK* form dsRNA that is recognized by DCL2 and/or DCL4. The resulting sRNA is stabilized by HEN1 and loaded onto AGO1 for further downregulation of *CBF1* mRNA. At 4°C, increased transcriptional activity of *CBF1* and *SVK* leads to RNAPII collisions.

METHODS

Plant material, growth conditions, and biomass determination

For analysis of steady-state RNA levels, seeds were surface sterilized with ethanol, stratified for 3 days, and grown on $^{1}/_{2}$ MS medium for 12 days in a long-day growth regimen (16 h light at 22°C/8 h dark 16°C and 100 μE). For short-day conditions, plants were grown on soil in a controlled manner (8 h light at 22°C/16 h dark 16°C and 100 μE) for 4 weeks. Biomass was measured after 4 weeks of SD growth and represents the wet weight. Mutants used in this study are in the Columbia background (Col-0) and have been previously described: *uns-1* (SALK_018442), *svk-1* (GABI_145A05), *SVK-OE* (SALK_007722) (Kindgren et al., 2018), *hen2-2* (GABI_774H07) (Lange et al., 2014), *sop2-1/rrp4-2* (Hématy et al., 2016), *dcl1-11* (Zhang et al., 2008), *dcl2dcl4* (SALK_064627/GABI_160G05) (Xie et al., 2005), *dcl3-1* (SALK_005512) (Xie et al., 2004), *ago1-36* (SALK_087076) (Baumberger and Baulcombe, 2005), *ago1-45* (Smith et al., 2009), *rdr1-1rdr2-1rdr6-15* (SAIL_672_F11, SAIL_1277_H08, SAIL_617_H07) (Garcia-Ruiz et al., 2010), *hen1-6* (SALK_090960) (Tang et al., 2012), *nrpd1-4* (SALK_083051) (Pontier et al., 2005), *nrpd1b-11* (SALK_029919) (Pontier et al., 2005), and *cbf3-1* (SAIL_244_D02) (Khanna et al., 2006). The *cbf1-3* (WiscDxLox504E12) and *cbf2-2* (SALK_067966) mutants are described in this study. Luciferase constructs used in this study have been described previously (Kindgren et al., 2018). Stable homozygous lines with one insertion were used for experiments.

RNA extraction and RT-qPCR

RNA was extracted with the EZNA Plant RNA Kit (Omega Bio-tek) according to the manufacturer's instructions. Total RNA was DNase-treated with Turbo DNase (Ambion). For cDNA synthesis, 1 μg of total RNA was used for reverse transcription (iScript, BioRad). For detection of *CBF1* and *SVK*, cDNA synthesis was performed with gene-specific primers with a tag and Superscript IV (Invitrogen) to ensure strand specificity. qPCR reactions were performed with iTaq Universal SYBR Green Supermix (BioRad) in 384-well plates. qPCR was performed in a CFX384 Touch Real-Time PCR Detection System (BioRad) and monitored with CFX Manager software (BioRad). Threshold values were subsequently exported to Excel (Microsoft Office) for further analysis. For each sample, three independent biological replicates with two to three technical replicates each were used. Two internal reference genes (*ACT2* [*At3g18780*] and *UBQ10* [*At4g05320*]) were used for relative gene expression calculations. All primers used in this study can be found in Supplemental Table 2.

Transcript stability assay

The half-life ($t_{1/2}$) of transcripts was determined as described previously (Fedak et al., 2016). In brief, Col-0 (wild-type) seedlings were grown vertically on plates containing $^{1}/_{2}$ MS medium for 10 days and then transferred to $^{1}/_{2}$ MS liquid medium and maintained at 22°C or shifted to 4°C for 4 h under the same light conditions. Seedlings from both temperatures were transferred into 12-well plates and incubated in buffer A (1 mM PIPES at pH 6.25, 1 mM trisodium citrate, 1 mM KCl, and 15 mM sucrose) at 22°C or 4°C, respectively. After 30 min of incubation, 150 mg/L cordycepin (3′-deoxyadenosine, Sigma Aldrich) was added and vacuum-infiltrated twice for 5 min. Samples were collected after 0, 15, 30, 60, 120, and 300 min in triplicate (15 seedlings per replicate), followed by total RNA extraction and RT-qPCR analysis using gene-specific primers (Supplemental Table 1). *EIF4A1* and *EXPL1* were used as assay controls (Perea-Resa et al., 2012; Fedak et al., 2016). C_t values were normalized with the C_t value at 0 min [$Ct(n) = (\ln(C_t/C_t(0)) \times (-10)$] and the slope to determine the half-life of the transcripts was calculated as follows [$t_{1/2} = (\ln_2)/\text{slope}$] of transcript.

Luciferase activity assay

CBF1 reporter constructs with or without the *SVK* gene unit at the 3′ end of *CBF1* were used as described previously to analyze the effect of *SVK* transcription on *CBF1* transcriptional output (Kindgren et al., 2018). In brief, two independent *CBF1* reporter constructs were used in the analysis. The *$_p$CBF1::CBF1:Tnos* construct carried the *CBF1* promoter) and *CBF1* gene body (−1903 bp to +639 bp relative to the translation start site) fused to the firefly luciferase gene (*LUC*) and the *Tnos* terminator. In the second construct, *$_p$CBF1::CBF1:LUC:SVK*, *Tnos* was replaced by the *SVK* promoter and transcription unit (+643 bp to +3410 bp relative to the *CBF1* translation start site) after removal of the stop codon in the *CBF1* ORF.

To perform the bioluminescent firefly luciferase assay, T3 homozygous seeds from two independent reporter lines for each construct were sterilized with bleach and stratified at 4°C for 2 days, followed by growth on $^{1}/_{2}$ MS medium in vertical plates under long-day conditions (16 h light/8 h dark) with 22°C/18°C day/night temperatures. Sufficient numbers (15–20) of 12-day-old individual seedlings in replicates were carefully placed and submerged in 96-well plates containing 200 μL luciferase assay reagent (5 mM luciferin in 0.01% Triton X-100). After a short 30-min incubation, plates were read using a GloMax Navigator Microplate Luminometer (Promega), and levels of bioluminescence produced per well were recorded. Data were exported, normalized for background bioluminescence noise from the average signal intensity of at least 4–5 wild-type *Col-0* seedlings with no LUC gene, and further analyzed to obtain the average LUC intensity per genotype.

dsRNA protection assay and dsRNA-IP-qPCR

The dsRNA protection assay was performed as described previously (Okano et al., 2014) with a few modifications. In brief, extracted total RNA (5 μg) was treated with DNase I and incubated with the indicated concentrations of RNase One Ribonuclease (Promega) for 1 h at 37°C. RNase-treated RNA was used as a template for reverse transcription using random primers. The resulting cDNA was used in a qPCR with oligos found in Supplemental Table 2. The data were further analyzed in Microsoft Excel, and relative steady-state levels were calculated by comparison with the level of *ACT2* in samples without RNase treatment. For dsRNA-IP-qPCR, nuclei from 3 g of 12-day-old seedlings were isolated with Honda buffer (0.44 M sucrose, 1.25% Ficoll, 2.5% Dextran T40, 20 mM Tris-HCl [pH 7.4], 10 mM $MgCl_2$, and 0.5% Triton-X). After addition of lysis buffer (0.3 M NaCl, 20 mM Tris-HCl [pH 7.4], 5 mM

$MgCl_2$, 5 mM BME, 0.5% Tween, and RNase and protease inhibitors) and centrifugation, the supernatant was mixed with a dsRNA-specific antibody (J2, Jena Biosciences), and IP was performed as described previously (Gao et al., 2020). The isolated RNA was used as a template in a room temperature reaction with Superscript IV (Invitrogen) and gene-specific oligos (Supplemental Table 2) and then used in qPCR reactions. IP samples were compared to input samples for further analysis.

Available sequencing data

Sequencing data from Kindgren et al. (plaNET-seq) (2019) and Schurch et al. (DRS-seq) (2014) were used in this study. Raw data were analyzed according to Kindgren et al. (2019). BedGraph files were opened in IGV (Broad Institute) and exported for further analysis. AGO1-interacting sRNAs were identified from published datasets (Liu et al., 2018; Annacondia and Martinez, 2021).

SUPPLEMENTAL INFORMATION

Supplemental information is available at *Plant Communications Online*.

FUNDING

This research was funded by the Swedish Research Council (2018–03926) and Carl Trygger Foundation (20:224).

AUTHOR CONTRIBUTIONS

V.Z., S.K., and P.K. conceived of and designed the study. V.Z., S.K., and P.K. undertook data collection. V.Z., S.K., and P.K. analyzed the data. All co-authors contributed to the writing of the manuscript.

ACKNOWLEDGMENTS

We thank members of the Kindgren lab for their critical reading of the manuscript. A special thanks to Dr. Germán Martinez-Arias for assistance with the AGO1-bound RNA data and the greenhouse personnel at Umeå Plant Science Centre for plant maintenance.

Received: December 12, 2022
Revised: January 17, 2023
Accepted: January 18, 2023
Published: January 21, 2023

REFERENCES

Achard, P., Gong, F., Cheminant, S., Alioua, M., Hedden, P., and Genschik, P. (2008). The cold-inducible CBF1 factor-dependent signaling pathway modulates the accumulation of the growth-repressing DELLA proteins via its effect on gibberellin metabolism. Plant Cell **20**:2117–2129. https://doi.org/10.1105/tpc.108.058941.

Alonso, J.M., Stepanova, A.N., Leisse, T.J., Kim, C.J., Chen, H., Shinn, P., Stevenson, D.K., Zimmerman, J., Barajas, P., Cheuk, R., et al. (2003). Genome-wide insertional mutagenesis of Arabidopsis thaliana. Science **301**:653–657. https://doi.org/10.1126/science.1086391.

Annacondia, M.L., and Martinez, G. (2021). Reprogramming of RNA silencing triggered by cucumber mosaic virus infection in Arabidopsis. Genome Biol. **22**:340. https://doi.org/10.1186/s13059-021-02564-z.

Badawi, M., Danyluk, J., Boucho, B., Houde, M., and Sarhan, F. (2007). The CBF gene family in hexaploid wheat and its relationship to the phylogenetic complexity of cereal CBFs. Mol. Genet. Genom. **277**:533–554. https://doi.org/10.1007/s00438-006-0206-9.

Baumberger, N., and Baulcombe, D.C. (2005). Arabidopsis ARGONAUTE1 is an RNA Slicer that selectively recruits microRNAs and short interfering RNAs. Proc. Natl. Acad. Sci. USA **102**:11928–11933. https://doi.org/10.1073/pnas.0505461102.

Benedict, C., Skinner, J.S., Meng, R., Chang, Y., Bhalerao, R., Huner, N.P.A., Finn, C.E., Chen, T.H.H., and Hurry, V. (2006). The CBF1-dependent low temperature signalling pathway, regulon and increase in freeze tolerance are conserved in Populus spp. Plant Cell Environ. **29**:1259–1272. https://doi.org/10.1111/j.1365-3040.2006.01505.x.

Bernstein, J., Patterson, D.N., Wilson, G.M., and Toth, E.A. (2008). Characterization of the essential activities of Saccharomyces cerevisiae Mtr4p, a helicase partner of the nuclear exosome. J. Biol. Chem. **283**:4930–4942. https://doi.org/10.1074/jbc.M706677200.

Bologna, N.G., and Voinnet, O. (2014). The diversity, biogenesis, and activities of endogenous silencing small RNAs in Arabidopsis. Annu. Rev. Plant Biol. **65**:473–503. https://doi.org/10.1146/annurev-arplant-050213-035728.

Borsani, O., Zhu, J., Verslues, P.E., Sunkar, R., and Zhu, J.-K. (2005). Endogenous siRNAs derived from a pair of natural cis-antisense transcripts regulate salt tolerance in Arabidopsis. Cell **123**:1279–1291. https://doi.org/10.1016/j.cell.2005.11.035.

Bouché, N., Lauressergues, D., Gasciolli, V., and Vaucheret, H. (2006). An antagonistic function for Arabidopsis DCL2 in development and a new function for DCL4 in generating viral siRNAs. EMBO J. **25**:3347–3356. https://doi.org/10.1038/sj.emboj.7601217.

Britto-Kido, S.d.A., Ferreira Neto, J.R.C., Pandolfi, V., Marcelino-Guimarães, F.C., Nepomuceno, A.L., Vilela Abdelnoor, R., Benko-Iseppon, A.M., and Kido, E.A. (2013). Natural antisense transcripts in plants: a review and identification in soybean infected with phakopsora pachyrhizi SuperSAGE library. Sci. World J. **2013**:219798. https://doi.org/10.1155/2013/219798.

Deleris, A., Gallego-Bartolome, J., Bao, J., Kasschau, K.D., Carrington, J.C., and Voinnet, O. (2006). Hierarchical action and inhibition of plant dicer-like proteins in antiviral defense. Science **313**:68–71. https://doi.org/10.1126/science.1128214.

Doherty, C.J., Van Buskirk, H.A., Myers, S.J., and Thomashow, M.F. (2009). Roles for Arabidopsis CAMTA transcription factors in cold-regulated gene expression and freezing tolerance. Plant Cell **21**:972–984. https://doi.org/10.1105/tpc.108.063958.

Dong, X., Yan, Y., Jiang, B., Shi, Y., Jia, Y., Cheng, J., Shi, Y., Kang, J., Li, H., Zhang, D., et al. (2020). The cold response regulator CBF1 promotes Arabidopsis hypocotyl growth at ambient temperatures. EMBO J. **39**:e103630. https://doi.org/10.15252/embj.2019103630.

Fang, X., Wang, L., Ishikawa, R., Li, Y., Fiedler, M., Liu, F., Calder, G., Rowan, B., Weigel, D., Li, P., and Dean, C. (2019). Arabidopsis FLL2 promotes liquid–liquid phase separation of polyadenylation complexes. Nature **569**:265–269. https://doi.org/10.1038/s41586-019-1165-8.

Fedak, H., Palusinska, M., Krzyczmonik, K., Brzezniak, L., Yatusevich, R., Pietras, Z., Kaczanowski, S., and Swiezewski, S. (2016). Control of seed dormancy in Arabidopsis by a cis-acting noncoding antisense transcript. Proc. Natl. Acad. Sci. USA **113**:E7846–E7855. https://doi.org/10.1073/pnas.1608827113.

Feng, L., Zhang, F., Zhang, H., Zhao, Y., Meyers, B.C., and Zhai, J. (2019). An online database for exploring over 2,000 Arabidopsis small RNA Libraries1 [OPEN]. Plant Physiol. **182**:685–691. https://doi.org/10.1104/pp.19.00959.

Fowler, S.G., Cook, D., and Thomashow, M.F. (2005). Low temperature induction of Arabidopsis CBF1, 2, and 3 is gated by the circadian clock. Plant Physiol. **137**:961–968. https://doi.org/10.1104/pp.104.058354.

Gangappa, S.N., Berriri, S., and Kumar, S.V. (2017). PIF4 coordinates thermosensory growth and immunity in Arabidopsis. Curr. Biol. **27**:243–249. https://doi.org/10.1016/j.cub.2016.11.012.

Gao, Y., Vasic, R., Song, Y., Teng, R., Liu, C., Gbyli, R., Biancon, G., Nelakanti, R., Lobben, K., Kudo, E., et al. (2020). m6A modification prevents formation of endogenous double-stranded RNAs and deleterious innate immune responses during hematopoietic development. Immunity **52**:1007–1021.e8. https://doi.org/10.1016/j.immuni.2020.05.003.

Garcia-Ruiz, H., Takeda, A., Chapman, E.J., Sullivan, C.M., Fahlgren, N., Brempelis, K.J., and Carrington, J.C. (2010). Arabidopsis RNA-dependent RNA polymerases and dicer-like proteins in antiviral defense and small interfering RNA biogenesis during turnip mosaic virus infection. Plant Cell **22**:481–496. https://doi.org/10.1105/tpc.109.073056.

Gilmour, S.J., Fowler, S.G., and Thomashow, M.F. (2004). Arabidopsis transcriptional activators CBF1, CBF2, and CBF3 have matching functional activities. Plant Mol. Biol. **54**:767–781. https://doi.org/10.1023/B:PLAN.0000040902.06881.d4.

Hématy, K., Bellec, Y., Podicheti, R., Bouteiller, N., Anne, P., Morineau, C., Haslam, R.P., Beaudoin, F., Napier, J.A., Mockaitis, K., et al. (2016). The zinc-finger protein SOP1 is required for a subset of the nuclear exosome functions in Arabidopsis. PLoS Genet. **12**:e1005817. https://doi.org/10.1371/journal.pgen.1005817.

Henriques, R., Wang, H., Liu, J., Boix, M., Huang, L.-F., and Chua, N.-H. (2017). The antiphasic regulatory module comprising CDF5 and its antisense RNA FLORE links the circadian clock to photoperiodic flowering. New Phytol. **216**:854–867. https://doi.org/10.1111/nph.14703.

Ito, Y., Katsura, K., Maruyama, K., Taji, T., Kobayashi, M., Seki, M., Shinozaki, K., and Yamaguchi-Shinozaki, K. (2006). Functional analysis of rice DREB1/CBF-type transcription factors involved in cold-responsive gene expression in transgenic rice. Plant Cell Physiol. **47**:141–153. https://doi.org/10.1093/pcp/pci230.

Jabnoune, M., Secco, D., Lecampion, C., Robaglia, C., Shu, Q., and Poirier, Y. (2013). A rice cis-natural antisense RNA acts as a translational enhancer for its cognate mRNA and contributes to phosphate homeostasis and plant fitness. Plant Cell **25**:4166–4182. https://doi.org/10.1105/tpc.113.116251.

Jaglo-Ottosen, K.R., Gilmour, S.J., Zarka, D.G., Schabenberger, O., and Thomashow, M.F. (1998). Arabidopsis CBF1 overexpression induces COR genes and enhances freezing tolerance. Science **280**:104–106. https://doi.org/10.1126/science.280.5360.104.

Jiang, B., Shi, Y., Peng, Y., Jia, Y., Yan, Y., Dong, X., Li, H., Dong, J., Li, J., Gong, Z., et al. (2020). Cold-induced CBF–PIF3 interaction enhances freezing tolerance by stabilizing the phyB thermosensor in Arabidopsis. Mol. Plant **13**:894–906. https://doi.org/10.1016/j.molp.2020.04.006.

Khanna, R., Shen, Y., Toledo-Ortiz, G., Kikis, E.A., Johannesson, H., Hwang, Y.-S., and Quail, P.H. (2006). Functional profiling reveals that only a small number of phytochrome-regulated early-response genes in Arabidopsis are necessary for optimal deetiolation. Plant Cell **18**:2157–2171. https://doi.org/10.1105/tpc.106.042200.

Kindgren, P., Ivanov, M., and Marquardt, S. (2019). Native elongation transcript sequencing reveals temperature dependent dynamics of nascent RNAPII transcription in Arabidopsis. Nucleic Acids Res. **48**:2332–2347. https://doi.org/10.1093/nar/gkz1189.

Kindgren, P., Ard, R., Ivanov, M., and Marquardt, S. (2018). Transcriptional read-through of the long non-coding RNA SVALKA governs plant cold acclimation. Nat. Commun. **9**:4561. https://doi.org/10.1038/s41467-018-07010-6.

Lange, H., Zuber, H., Sement, F.M., Chicher, J., Kuhn, L., Hammann, P., Brunaud, V., Bérard, C., Bouteiller, N., Balzergue, S., et al. (2014). The RNA helicases AtMTR4 and HEN2 target specific subsets of nuclear transcripts for degradation by the nuclear exosome in Arabidopsis thaliana. PLoS Genet. **10**:e1004564. https://doi.org/10.1371/journal.pgen.1004564.

Lapidot, M., and Pilpel, Y. (2006). Genome-wide natural antisense transcription: coupling its regulation to its different regulatory mechanisms. EMBO Rep. **7**:1216–1222. https://doi.org/10.1038/sj.embor.7400857.

Li, S., Liberman, L.M., Mukherjee, N., Benfey, P.N., and Ohler, U. (2013). Integrated detection of natural antisense transcripts using strand-specific RNA sequencing data. Genome Res. **23**:1730–1739. https://doi.org/10.1101/gr.149310.112.

Liu, C., Xin, Y., Xu, L., Cai, Z., Xue, Y., Liu, Y., Xie, D., Liu, Y., and Qi, Y. (2018). Arabidopsis ARGONAUTE 1 binds chromatin to promote gene transcription in response to hormones and stresses. Dev. Cell **44**:348–361.e7. https://doi.org/10.1016/j.devcel.2017.12.002.

Liu, F., Marquardt, S., Lister, C., Swiezewski, S., and Dean, C. (2010). Targeted 3' processing of antisense transcripts triggers Arabidopsis FLC chromatin silencing. Science **327**:94–97. https://doi.org/10.1126/science.1180278.

Lubas, M., Christensen, M.S., Kristiansen, M.S., Domanski, M., Falkenby, L.G., Lykke-Andersen, S., Andersen, J.S., Dziembowski, A., and Jensen, T.H. (2011). Interaction profiling identifies the human nuclear exosome targeting complex. Mol. Cell **43**:624–637. https://doi.org/10.1016/j.molcel.2011.06.028.

Medina, J., Bargues, M., Terol, J., Pérez-Alonso, M., and Salinas, J. (1999). The Arabidopsis CBF gene family is composed of three genes encoding AP2 domain-containing proteins whose expression is regulated by low temperature but not by abscisic acid or dehydration. Plant Physiol. **119**:463–470. https://doi.org/10.1104/pp.119.2.463.

Meena, S.K., Heidecker, M., Engelmann, S., Jaber, A., de Vries, T., Baumann-Kaschig, K., Abel, S., Behrens, S.-E., and Gago-Zachert, S. (2021). Expression levels of long noncoding natural antisense transcripts overlapping the UGT73C6 gene affect rosette size of Arabidopsis thaliana. Preprint at bioRxiv. https://doi.org/10.1101/2021.03.01.433436.

Nieto, C., López-Salmerón, V., Davière, J.M., and Prat, S. (2015). ELF3-PIF4 interaction regulates plant growth independently of the evening complex. Curr. Biol. **25**:187–193. https://doi.org/10.1016/j.cub.2014.10.070.

Norén, L., Kindgren, P., Stachula, P., Rühl, M., Eriksson, M.E., Hurry, V., and Strand, Å. (2016). Circadian and plastid signaling pathways are integrated to ensure correct expression of the *CBF* and *COR* genes during photoperiodic growth. Plant Physiol. **171**:1392–1406. https://doi.org/10.1104/pp.16.00374.

Okano, Y., Senshu, H., Hashimoto, M., Neriya, Y., Netsu, O., Minato, N., Yoshida, T., Maejima, K., Oshima, K., Komatsu, K., et al. (2014). In planta recognition of a double-stranded RNA synthesis protein complex by a potexviral RNA silencing suppressor the plant cell. Plant Cell **26**:2168–2183. https://doi.org/10.1105/tpc.113.120535.

Perea-Resa, C., Hernández-Verdeja, T., López-Cobollo, R., del Mar Castellano, M., and Salinas, J. (2012). LSM proteins provide accurate splicing and decay of selected transcripts to ensure normal Arabidopsis development. Plant Cell **24**:4930–4947. https://doi.org/10.1105/tpc.112.103697.

Pontier, D., Yahubyan, G., Vega, D., Bulski, A., Saez-Vasquez, J., Hakimi, M.-A., Lerbs-Mache, S., Colot, V., and Lagrange, T. (2005). Reinforcement of silencing at transposons and highly repeated sequences requires the concerted action of two distinct RNA polymerases IV in Arabidopsis. Genes Dev. **19**:2030–2040. https://doi.org/10.1101/gad.348405.

Reis, R.S., and Poirier, Y. (2021). Making sense of the natural antisense transcript puzzle. Trends Plant Sci. **26**:1104–1115. https://doi.org/10.1016/j.tplants.2021.07.004.

Rosa, S., Duncan, S., and Dean, C. (2016). Mutually exclusive sense–antisense transcription at FLC facilitates environmentally induced gene repression. Nat. Commun. **7**:13031. https://doi.org/10.1038/ncomms13031.

Schurch, N.J., Cole, C., Sherstnev, A., Song, J., Duc, C., Storey, K.G., McLean, W.H.I., Brown, S.J., Simpson, G.G., and Barton, G.J.

(2014). Improved annotation of 3′ untranslated regions and complex loci by combination of strand-specific direct RNA sequencing, RNA-seq and ESTs. PLoS One **9**:e94270. https://doi.org/10.1371/journal.pone.0094270.

Shepherd, T.G. (2016). Effects of a warming arctic. Science **353**:989–990. https://doi.org/10.1126/science.aag2349.

Smith, M.R., Willmann, M.R., Wu, G., Berardini, T.Z., Möller, B., Weijers, D., and Poethig, R.S. (2009). Cyclophilin 40 is required for microRNA activity in Arabidopsis. Proc. Natl. Acad. Sci. USA **106**:5424–5429. https://doi.org/10.1073/pnas.0812729106.

Sorenson, R.S., Deshotel, M.J., Johnson, K., Adler, F.R., and Sieburth, L.E. (2018). Arabidopsis mRNA decay landscape arises from specialized RNA decay substrates, decapping-mediated feedback, and redundancy. Proc. Natl. Acad. Sci. USA **115**:E1485–E1494. https://doi.org/10.1073/pnas.1712312115.

Swiezewski, S., Liu, F., Magusin, A., and Dean, C. (2009). Cold-induced silencing by long antisense transcripts of an Arabidopsis Polycomb target. Nature **462**:799–802. https://doi.org/10.1038/nature08618.

Tang, X., Bian, S., Tang, M., Lu, Q., Li, S., Liu, X., Tian, G., Nguyen, V., Tsang, E.W.T., Wang, A., et al. (2012). MicroRNA–mediated repression of the seed maturation program during vegetative development in Arabidopsis. PLoS Genet. **8**:e1003091. https://doi.org/10.1371/journal.pgen.1003091.

Thieffry, A., Vigh, M.L., Bornholdt, J., Ivanov, M., Brodersen, P., and Sandelin, A. (2020). Characterization of Arabidopsis thaliana promoter bidirectionality and antisense RNAs by inactivation of nuclear RNA decay pathways. Plant Cell **32**:1845–1867. https://doi.org/10.1105/tpc.19.00815.

Thomashow, M.F. (1999). Plant cold acclimation: freezing tolerance genes and regulatory mechanisms. Annu. Rev. Plant Physiol. Plant Mol. Biol. **50**:571–599. https://doi.org/10.1146/annurev.arplant.50.1.571.

Tian, B., and Manley, J.L. (2017). Alternative polyadenylation of mRNA precursors. Nat. Rev. Mol. Cell Biol. **18**:18–30. https://doi.org/10.1038/nrm.2016.116.

Wang, H., Chung, P.J., Liu, J., Jang, I.-C., Kean, M.J., Xu, J., and Chua, N.-H. (2014). Genome-wide identification of long noncoding natural antisense transcripts and their responses to light in Arabidopsis. Genome Res. **24**:444–453. https://doi.org/10.1101/gr.165555.113.

Wang, X.-J., Gaasterland, T., and Chua, N.-H. (2005). Genome-wide prediction and identification of cis-natural antisense transcripts in Arabidopsis thaliana. Genome Biol. **6**:R30. https://doi.org/10.1186/gb-2005-6-4-r30.

Wunderlich, M., Gross-Hardt, R., and Schöffl, F. (2014). Heat shock factor HSFB2a involved in gametophyte development of Arabidopsis thaliana and its expression is controlled by a heat-inducible long non-coding antisense RNA. Plant Mol. Biol. **85**:541–550. https://doi.org/10.1007/s11103-014-0202-0.

Xie, Z., Allen, E., Wilken, A., and Carrington, J.C. (2005). DICER-LIKE 4 functions in trans-acting small interfering RNA biogenesis and vegetative phase change in *Arabidopsis thaliana*. Proc. Natl. Acad. Sci. USA **102**:12984–12989. https://doi.org/10.1073/pnas.0506426102.

Xie, Z., Johansen, L.K., Gustafson, A.M., Kasschau, K.D., Lellis, A.D., Zilberman, D., Jacobsen, S.E., and Carrington, J.C. (2004). Genetic and functional diversification of small RNA pathways in plants. PLoS Biol. **2**:e104. https://doi.org/10.1371/journal.pbio.0020104.

Yuan, C., Wang, J., Harrison, A.P., Meng, X., Chen, D., and Chen, M. (2015). Genome-wide view of natural antisense transcripts in Arabidopsis thaliana. DNA Res. **22**:233–243. https://doi.org/10.1093/dnares/dsv008.

Zarka, D.G., Vogel, J.T., Cook, D., and Thomashow, M.F. (2003). Cold induction of Arabidopsis CBF genes involves multiple ICE (inducer of CBF expression) promoter elements and a cold-regulatory circuit that is desensitized by low temperature. Plant Physiol. **133**:910–918. https://doi.org/10.1104/pp.103.027169.

Zhang, J.-F., YUAN, L.-J., SHAO, Y., DU, W., YAN, D.-W., and LU, Y.-T. (2008). The disturbance of small RNA pathways enhanced abscisic acid response and multiple stress responses in Arabidopsis. Plant Cell Environ. **31**:562–574. https://doi.org/10.1111/j.1365-3040.2008.01786.x.

Zhang, X., Lii, Y., Wu, Z., Polishko, A., Zhang, H., Chinnusamy, V., Lonardi, S., Zhu, J.-K., Liu, R., and Jin, H. (2013). Mechanisms of small RNA generation from cis-NATs in response to environmental and developmental cues. Mol. Plant **6**:704–715. https://doi.org/10.1093/mp/sst051.

Zhang, X., Xia, J., Lii, Y.E., Barrera-Figueroa, B.E., Zhou, X., Gao, S., Lu, L., Niu, D., Chen, Z., Leung, C., et al. (2012). Genome-wide analysis of plant nat-siRNAs reveals insights into their distribution, biogenesis and function. Genome Biol. **13**:R20. https://doi.org/10.1186/gb-2012-13-3-r20.

Zhang, Y.-J., Yang, J.-S., Guo, S.-J., Meng, J.-J., Zhang, Y.-L., Wan, S.-B., He, Q.-W., and Li, X.-G. (2011). Over-expression of the Arabidopsis CBF1 gene improves resistance of tomato leaves to low temperature under low irradiance. Plant Biol. **13**:362–367. https://doi.org/10.1111/j.1438-8677.2010.00365.x.

Plant Communications, Volume *4*

Supplemental information

The non-coding RNA *SVALKA* locus produces a *cis*-natural antisense transcript that negatively regulates the expression of *CBF1* and bio-mass production at normal temperatures

Vasiliki Zacharaki, Shiv Kumar Meena, and Peter Kindgren

Supplementary Information

Zacharaki et al.

Supplementary Figure 1-4

Supplementary Table 1: AGO1 small RNAs

Supplementary Table 2: Oligos used in this study

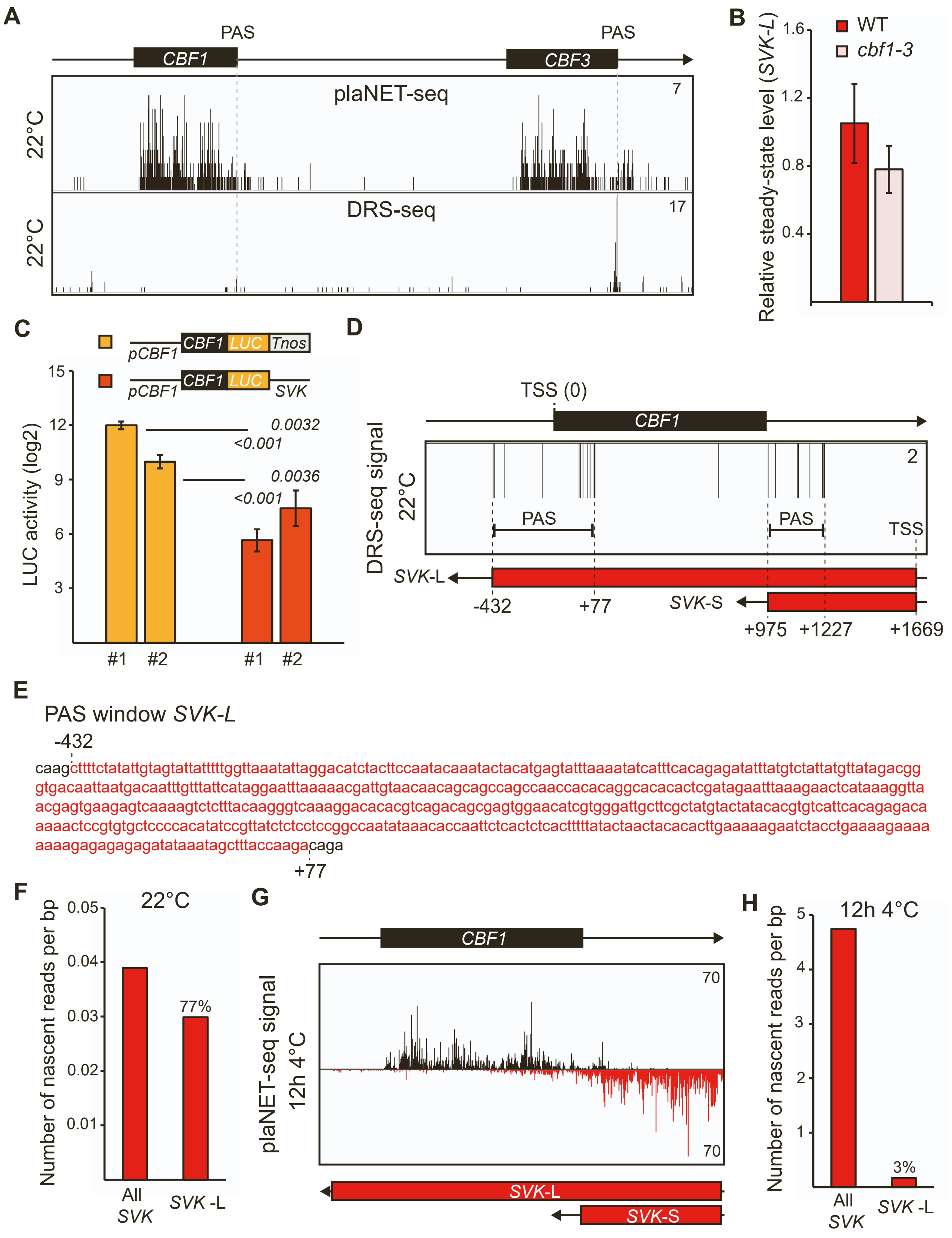
A
PAS
PAS
CBF1
CBF3
22°C
plaNET-seq
7
22°C
DRS-seq
17
B
Relative steady-state level (SVK-L)
1.6
1.2
0.8
0.4
WT
cbf1-3
C
pCBF1 CBF1 LUC Tnos
pCBF1 CBF1 LUC SVK
LUC activity (log2)
15
12
9
6
3
0.0032
<0.001
0.0036
<0.001
#1
#2
#1
#2
D
TSS (0)
CBF1
DRS-seq signal
22°C
2
PAS
PAS
TSS
SVK-L
SVK-S
-432
+77
+975
+1227
+1669
E
PAS window SVK-L
-432
caagcttttctatattgtagtattatttttggttaaatattaggacatctacttccaatacaaatactacatgagtatttaaaatatcatttcacagagatatttatgtctattatgttatagacgg
gtgacaattaatgacaatttgtttattcataggaatttaaaaacgattgtaacaacagcagccagccaaccacacaggcacacactcgatagaatttaaagaactcataaaggtta
acgagtgaagagtcaaaagtctctttacaagggtcaaaggacacacgtcagacagcgagtggaacatcgtgggattgcttcgctatgtactatacacgtgtcattcacagagaca
aaaactccgtgtgctccccacatatccgttatctctcctccggccaatataaacaccaattctcactctcactttttatactaactacacacttgaaaaagaatctacctgaaaagaaaa
aaaagagagagagagatataaatagctttaccaagacaga
+77
F
22°C
Number of nascent reads per bp
0.05
0.04
0.03
0.02
0.01
77%
All SVK
SVK -L
G
CBF1
plaNET-seq signal
12h 4°C
70
70
SVK-L
SVK-S
H
12h 4°C
Number of nascent reads per bp
5
4
3
2
1
3%
All SVK
SVK -L

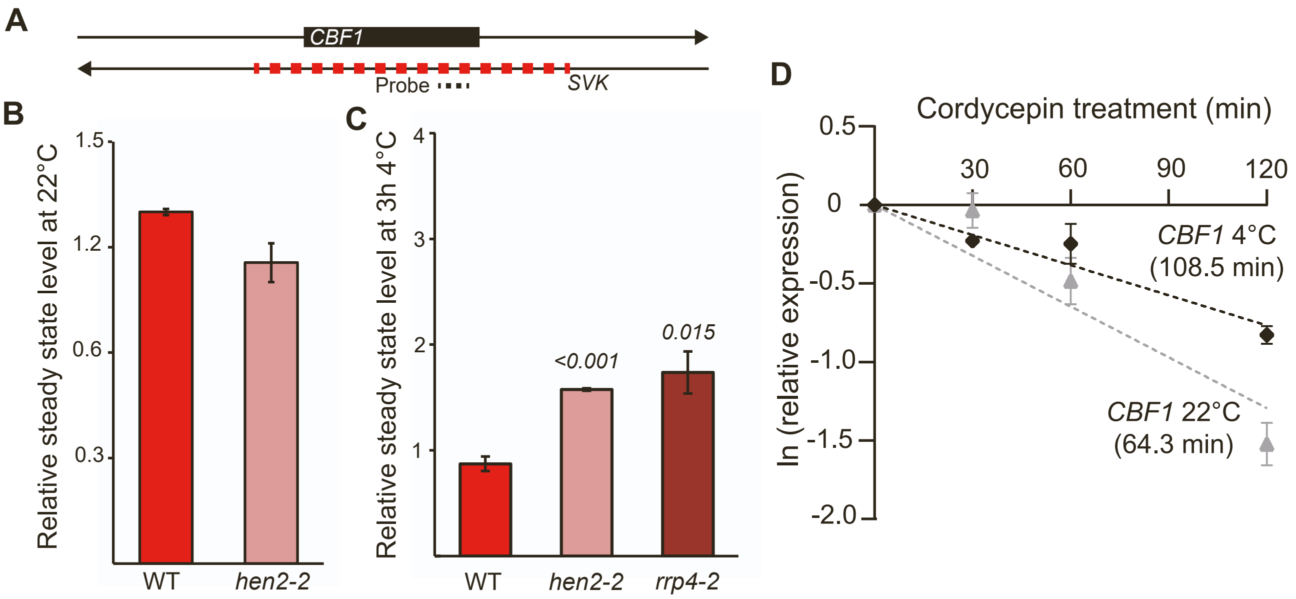
A
CBF1
Probe
SVK
B
Relative steady state level at 22°C
1.5
1.2
0.6
0.3
WT
hen2-2
C
Relative steady state level at 3h 4°C
4
3
2
1
<0.001
0.015
WT
hen2-2
rrp4-2
D
Cordycepin treatment (min)
30
60
90
120
0.5
0
-0.5
-1.0
-1.5
-2.0
ln (relative expression)
CBF1 4°C
(108.5 min)
CBF1 22°C
(64.3 min)

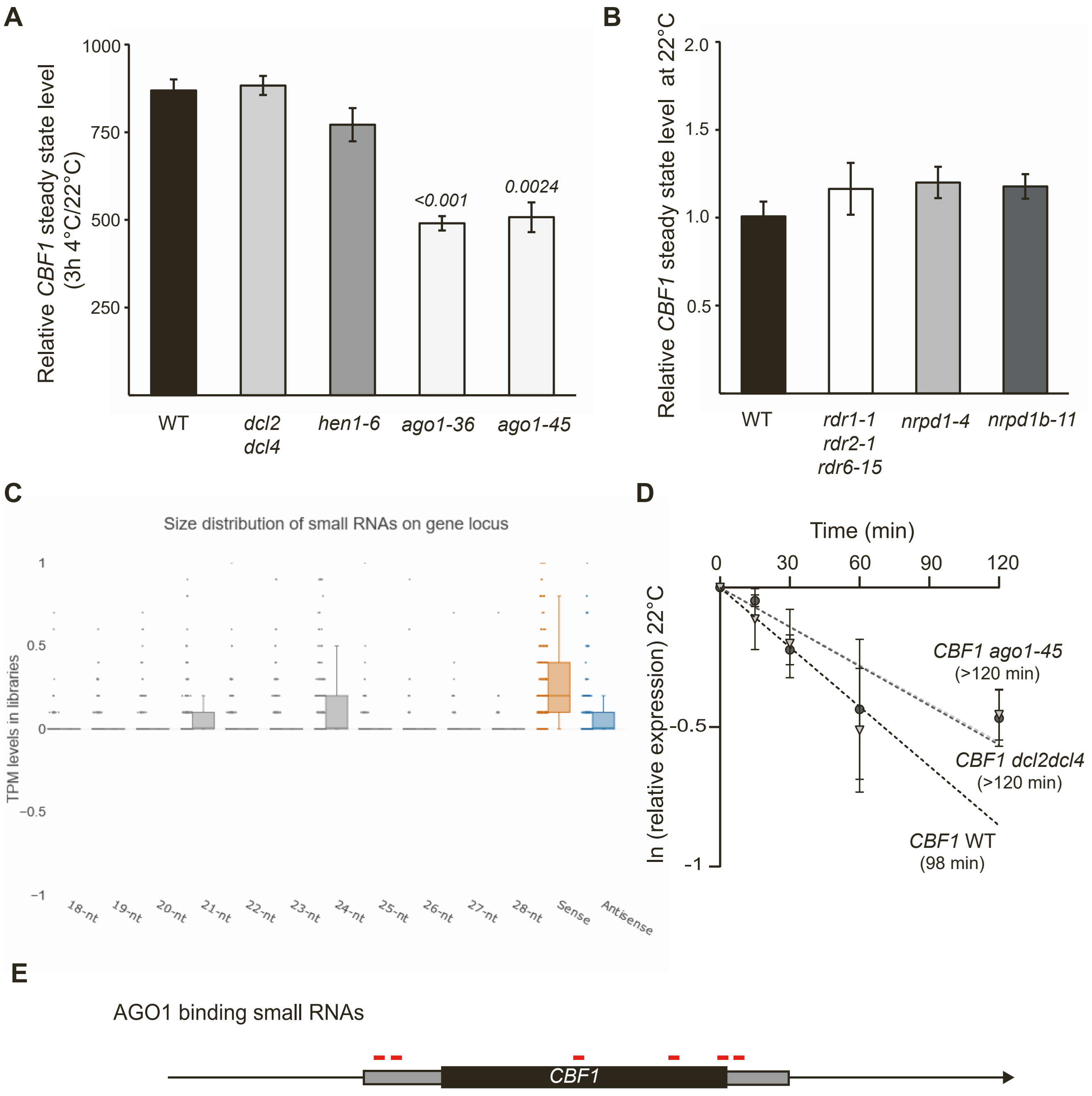
A
Relative CBF1 steady state level (3h 4°C/22°C)
1000
750
500
250
<0.001
0.0024
WT
dcl2 dcl4
hen1-6
ago1-36
ago1-45
B
Relative CBF1 steady state level at 22°C
2.0
1.5
1.0
0.5
WT
rdr1-1 rdr2-1 rdr6-15
nrpd1-4
nrpd1b-11
C
Size distribution of small RNAs on gene locus
TPM levels in libraries
1
0.5
0
−0.5
−1
18-nt
19-nt
20-nt
21-nt
22-nt
23-nt
24-nt
25-nt
26-nt
27-nt
28-nt
Sense
Antisense
D
Time (min)
0
30
60
90
120
ln (relative expression) 22°C
-0.5
-1
CBF1 ago1-45
(>120 min)
CBF1 dcl2dcl4
(>120 min)
CBF1 WT
(98 min)
E
AGO1 binding small RNAs
CBF1

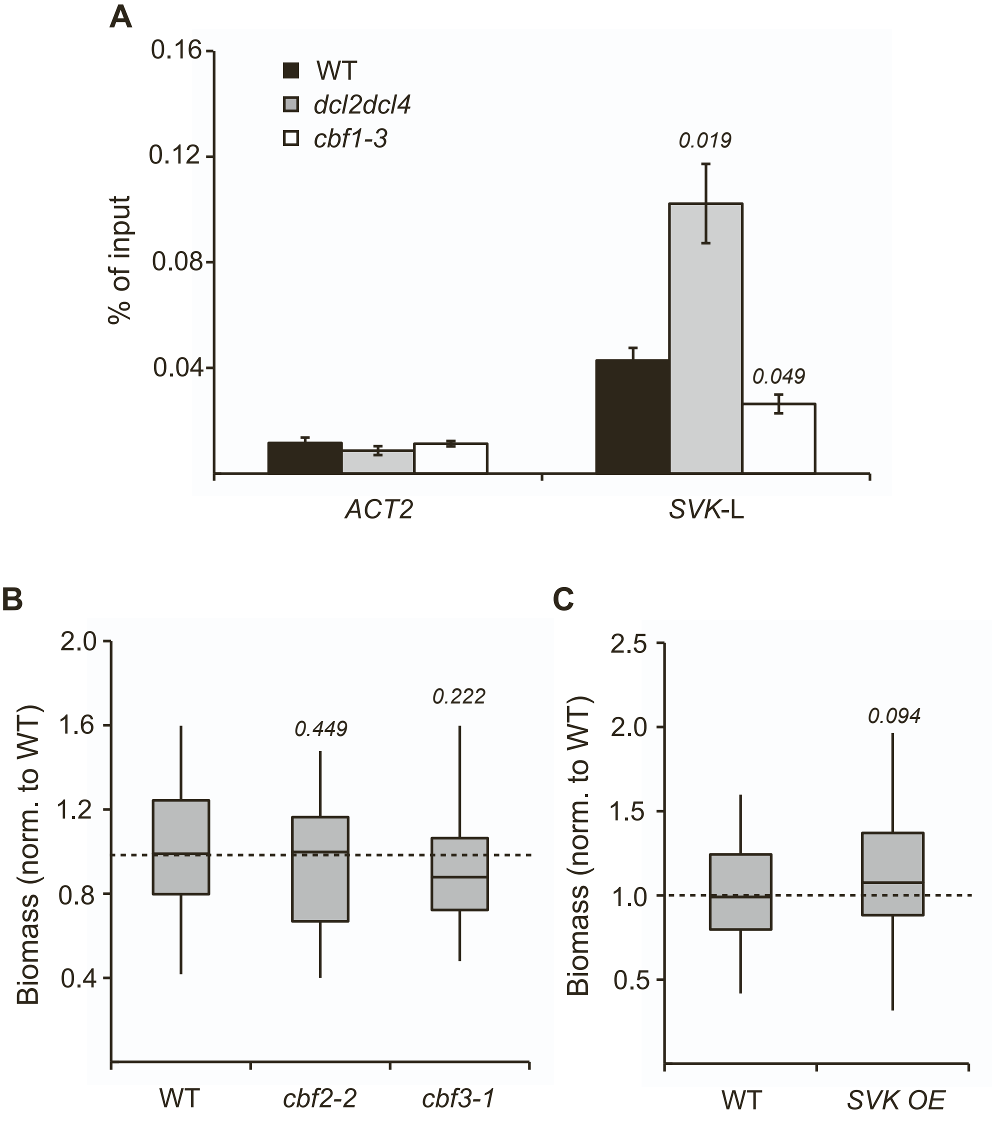

A
0.16
0.12
0.08
0.04
% of input
WT
dcl2dcl4
cbf1-3
0.019
0.049
ACT2
SVK-L
B
2.0
1.6
1.2
0.8
0.4
Biomass (norm. to WT)
0.449
0.222
WT
cbf2-2
cbf3-1
C
2.5
2.0
1.5
1.0
0.5
Biomass (norm. to WT)
0.094
WT
SVK OE

Supplementary Table 1

small RNAs binding to AGO1

**Sequence**
tgaaaaagaatctacc
aaaaaaagagagagagatataaa
ACTTCGCTGACTCGGCTTGGCGGCTACGAATCCCGG
TATATGGATGAGGAGACAATG
GAGTTACTAAtatt
tttccatttttgtactatagt

Supplementary Table 2

Oligos used in this study

For RT-qPCR

Locus	Name	Sequence (5´-3´)	Comment
-	rtTAG	GACTGGAGCACGAGGACACT	Oligo used in qPCR to amplify sequence from linker on RT primer
At4g25490	*CBF1*	GACTGGAGCACGAGGACACggtaaagctatttatatctctctctc	RT primer
		cacacttgaaaaagaatctacctg	Forward primer in qPCR
At4g07395	*SVK (probe c)*	GACTGGAGCACGAGGACACTacccgaaataaacaaatccgta	RT primer
		ggagaagcaagacgacaacg	Forward primer in qPCR
	SVK (probe b)	GACTGGAGCACGAGGACACTtcgatagtcgtttccatttttgt	RT primer
		aaaatgaaggaaccattctaaaaa	Forward primer in qPCR
	SVK (probe a)	GACTGGAGCACGAGGACACTcacacttgaaaaagaatctacctg	Reverse primer in qPCR, RT primer
		ggtaaagctatttatatctctctctc	Forward primer in qPCR
At3g18780	*ACT2*	CTTGCACCAAGCAGCATGAA	Reverse primer in qPCR, RT primer
		CCGATCCAGACACTGTACTTCCTT	Forward primer in qPCR
At4g05320	*UBQ10*	GGCCTTGTATAATCCCTGATGAATAAG	Reverse primer in qPCR, RT primer
		AAAGAGATAACAGGAACGGAAACATAGT	Forward primer in qPCR
At3g45970	*EXP*	CAAGTCGGTTCATCGCCAAATTGGG	Reverse primer in qPCR, RT primer
		GTATCCACCGGTTACTACGAACCTG	Forward primer in qPCR
At3g13920	*EIF4A*	GGCGTAAGGTTGATTGGCTCAC	Reverse primer in qPCR, RT primer
		GATGAGAACACGGGAGGAACCAG	Forward primer in qPCR
